# Space-time analysis of head and neck cancer in Asia and its 34 countries and territories (1990–2021): Implications from the Global Burden of Disease Study 2021

**DOI:** 10.1371/journal.pone.0326177

**Published:** 2025-06-17

**Authors:** Mengjuan Ding, Shengjian Yu, Yurong Chen, Yewen Liu, Feng Xuan

**Affiliations:** 1 Department of Quality Management, Zhuji Blood Bank, Shaoxing, Zhejiang Province, China; 2 Department of Radiation Oncology, Zhuji Affiliated Hospital of Wenzhou Medical University, Shaoxing, Zhejiang Province, China; 3 Department of Medical Oncology, Zhuji Affiliated Hospital of Wenzhou Medical University, Shaoxing, Zhejiang Province, China; 4 Department of Medical Laboratory, Zhuji Affiliated Hospital of Wenzhou Medical University, Shaoxing, Zhejiang Province, China; National center for chronic and non-communicable diesease prevention and control, CHINA

## Abstract

**Background:**

Asia bears a disproportionate burden of head and neck cancer (HNC). This study aimed to analyze its spatial distribution and temporal trends in Asia from 1990 to 2021, projecting trends to 2030.

**Methods:**

We performed a secondary analysis of data from the Global Burden of Disease Study (GBD) 2021, examining disability-adjusted life years (DALYs) for HNC and its five major subtypes: nasopharyngeal cancer (NPC), thyroid cancer (TC), laryngeal cancer (LC), lip and oral cavity cancer (LOC), and other pharyngeal cancer (OPC), across five Asian subregions and 34 countries/territories from 1990 to 2021. Temporal trends were evaluated using Joinpoint regression, and projections to 2030 were generated through Bayesian Age-Period-Cohort model.

**Results:**

From 1990 to 2021, DALYs for HNC increased in five subregions. In contrast, age-standardized DALY rates (ASDR) declined across all subregions except South Asia, with East Asia experiencing the most rapid decrease. In 2021, South Asia recorded the highest DALYs (6,412,639) and ASDR (405.82 per 100,000) for HNC. LOC was the main HNC type in most regions (32.41% − 46.23%), except East Asia, where NPC was most common (38.96%). South Asia also exhibited the highest ASDRs for LC (67.29), LOC (182.29), and OPC (93.00) per 100,000, while Southeast Asia demonstrated the highest ASDRs for NPC (50.77) and TC (18.22) per 100,000. Significant disparities in ASDR trends for HNC subtypes were observed across Asia. By 2030, South Asia is projected to maintain the highest ASDRs for HNC (394.59), LC (62.98), LOC (185.31), and OPC (95.50). East and Southeast Asia are expected to show comparable ASDRs for NPC (approximately 50.00), with Southeast Asia leading in TC ASDR (23.90).

**Conclusions:**

HNC remains a significant public health challenge in Asia, with substantial heterogeneity in its subtypes across the five subregions. Implementing targeted, region-specific strategies is crucial to mitigating the disease burden.

## Introduction

Head and neck cancer (HNC), including nasopharyngeal, laryngeal, oral, pharyngeal, and thyroid cancers, account for 9.4% (1,767,629 cases) of global cancer incidence and 5.5% (529,488 cases) of cancer-related deaths, as reported in the 2022 Global Cancer Statistics [1]. Lifestyle factors, including tobacco use, alcohol consumption, and human papillomavirus (HPV) infection, have been identified as risk factors of HNC in previous research [2]. Despite advancements in cancer treatment, the 5-year survival rate for HNC has only rose from 55% during 1992–1996 to 66% during 2002–2006 [3]. Furthermore, the global burden of HNC remained significantly imbalanced, with Asia bearing a disproportionately high share of these malignancies. In 2022, Asia accounted for 65.8% of global HNC cases and 72% of deaths, despite its 59.2% share of the world’s population [1]. Additionally, the epidemiological characteristics of HNC in Asia continue to change.

Global epidemiological studies of HNC have shown considerable progress to date. For example, M. Du et al. noted a substantial decline in the global incidence of nasopharyngeal cancer (**Estimated Annual Percentage Change **[EAPC ]= –1.52) from 1990 to 2017, while lip and oral cavity cancer (EAPC = 0.26) and other pharyngeal cancer (EAPC = 0.62) exhibited increasing trends [4]. Similarly, Supei Hu et al. demonstrated that the global age-standardized incidence rate (ASIR) of thyroid cancer increased from 2.01 to 2.83 per 100,000 population between 1990 and 2019, despite a decrease in the age-standardized disability-adjusted life years rate (ASDR) from 15.55 to 14.98 per 100,000 population [5]. Furthermore, Tianjiao Zhou et al. reported that in 1990, the worldwide ASIR was 13.97 per 100,000 (1,159,496 cases), and the ASDR was 186.27 per 100,000 (15,570,403 disability-adjusted life years [DALYs]). Between 1990 and 2021, the ASIR increased by 0.35% annually, while the ASDR decreased by 0.69% annually [6]. Notably, East Asia experienced the most rapid increase in ASIR among the 21 Global Burden of Disease (GBD) regions. However, in Asia, a region with a notable burden of HNC, systematic and detailed epidemiological studies are still lacking. This gap limits our ability to accurately assess public health needs and evaluate the effectiveness of prevention and control strategies.

To fill this gap, we conducted a secondary analysis of the burden of HNC in Asia using the most recent GBD 2021 data. The primary aim of this study was to comprehensively characterize the temporal trends and regional disparities in the burden of HNC and its subtypes from 1990 to 2030 across Asia. By applying the Joinpoint regression model, we identified changes in disease trends from 1990 to 2021, and projected these trends through 2030 with the Bayesian Age-Period-Cohort (BAPC) model. This space-time analysis was designed to generate evidence that not only reflects the evolving epidemiological patterns of HNC in Asia but also informs regional policymakers in developing targeted cancer control strategies.

## Methods

### Data sources

The 2021 GBD study is a comprehensive epidemiological research project that offers global data on 88 risk factors, 371 diseases and injuries, 288 causes of death from 1990 to 2021 [7–9]. It incorporates extensive data sources like vital registration systems, epidemiological surveys, disease surveillance systems, cancer registries, police records, and open-source databases. Three main standardized analytical tools were employed by the GBD 2021 Collaborators to estimate prevalence, incidence, and mortality. DisMod-MR 2.1 (Disease Modelling Meta-Regression; version 2.1), a Bayesian meta-regression tool, ensures internal consistency among epidemiological parameters by synthesizing heterogeneous data sources across time and geography. ST-GPR is used primarily for generating covariate estimates and smoothing trends by incorporating spatial and temporal dependencies. CODEm is designed to model cause-specific mortality by evaluating multiple predictive models and generating ensemble estimates based on out-of-sample predictive validity. Detailed descriptions of these methods can be found in prior academic literature [7–9].

In our secondary analysis, we focused on analyzing major head and neck cancers, including nasopharynx (NPC), thyroid (TC), larynx (LC), lip and oral cavity (LOC), and other pharynx cancers (OPC). The International Classification of Diseases (ICD) codes for these cancers, from both the Ninth and Tenth Revisions, were provided in S1 Table for reference. Drawing data from the 2021 GBD study, we identified DALYs as the indicator to characterize the disease burden. The DALYs, a composite measure that integrates years of life lost due to premature mortality and years lived with disability, offers a comprehensive assessment of disease burden. Accordingly, the rates associated with DALYs were presented per 100,000 population, accompanied by a 95% uncertainty interval as per the GBD methodology. 204 countries and territories are categorized into 21 GBD regions for data analysis purposes, based on epidemiological and geographical criteria [10]. In our study, the five Asia subregions, which include High-income Asia Pacific, East Asia, Southeast Asia, Central Asia, and South Asia, encompass 34 countries and territories (S2 Table).

### Statistical analysis

While 2021 GBD study only estimated the burden of five major head and neck cancers, we defined the overall burden of HNC by integrating data on nasopharynx, thyroid, larynx, lip and oral cavity, and other pharynx cancers [11]. As no published uncertainty intervals (UIs) were available for HNC estimates, we calculated 95% confidence intervals (CIs) based on the corresponding standard errors. These were determined by dividing the width of the 95% UI by 3.92 (1.96 × 2) [11]. The Joinpoint Regression Program, freely provided by the US National Cancer Institute Surveillance Research Program, was applied to analyze the temporal trends in age-standardized DALYs rate from 1990 to 2021 [12,13]. This program identifies the points in time (joinpoints) where there is a statistically significant change in the trend. We allowed for a maximum of five joinpoints and used the Monte Carlo permutation method to select the best model. The final model detailed the number of joinpoints throughout the study period, reporting the annual percent change (APC) and the accompanying 95% CI. A negative upper limit for both the APC and its 95% CI implies a significant downtrend. In contrast, a significant uptrend is denoted when the lower limit of the APC and its 95% CI is positive. Additionally, the model also calculates the average annual percentage change (AAPC) and its corresponding 95% CI, which provide a simplified overview of the trend throughout the past three decades. Lastly, the disease burden projection from 2022 to 2030 was conducted using the BAPC model [14]. The model integrates nested Laplace approximations (INLA) to facilitate full Bayesian inference, providing a more efficient and accurate prediction compared to traditional Markov chain Monte Carlo (MCMC) methods

Analyses were performed using R (version 4.4.1) and the Joinpoint Regression Program (version 5.1.0.0). Key packages utilized were “ggplot2”, “BAPC”, and “INLA”. A p-value of <0.05 was considered statistically significant.

### Ethics statement

Given the de-identified nature and public availability of the GBD data, this study is exempt from institutional ethical board review.

## Results

### Burden of head and neck cancer

**Fig 1:**
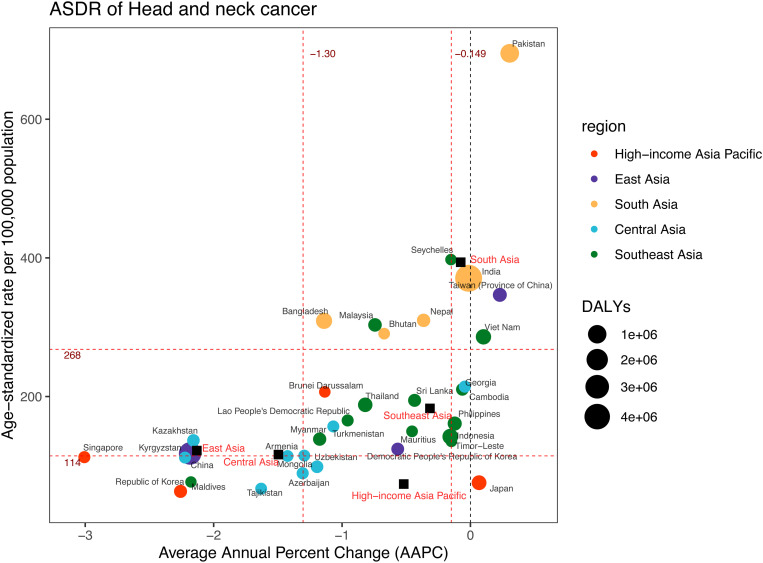
DALYs and ASDR of head and neck cancer in 2021, and their average annual percentage changes from 1990 to 2021. The black squares indicate the five Asia subregions. The circles’ colors identify the GBD region of each country or territory, and their sizes reflect the DALYs values. Red dashed lines indicate the tertiles of AAPC or ASDR (with corresponding values). ASDR = Age-standardised DALYs rate. DALYs = Disability-Adjusted Life Years. AAPC = Average annual percentage change.

**Fig 2:**
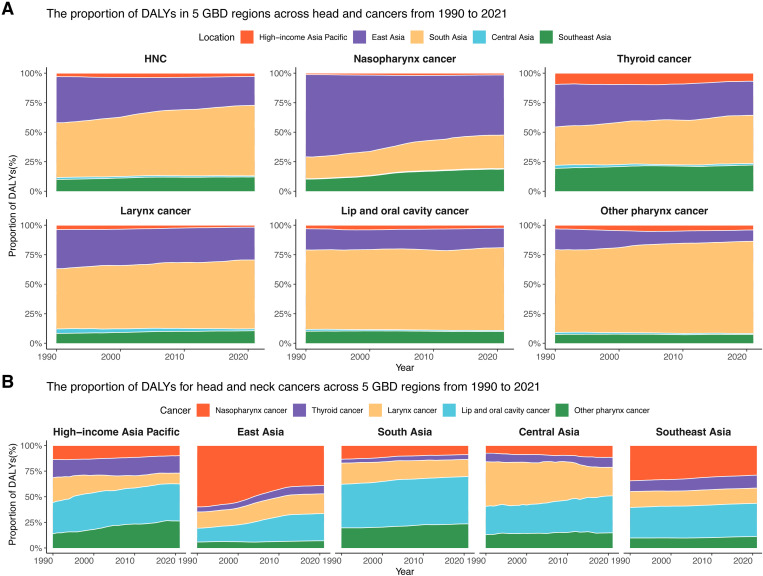
The percentage change in DALYs for head and neck cancers in the Asia between 1990 and 2021. (A) The proportion of DALYs in 5 GBD regions across head and cancers from 1990 to 2021. (B) The proportion of DALYs for head and neck cancer across 5 GBD regions from 1990 to 2021.

From 1990 to 2021, five subregions within Asia experienced an upward trend in DALYs associated with HNC (Table 1, S1 Fig). South Asia recorded the highest DALYs at approximately 6,412,639 (95%CI: 5,537,665–7,259,268) in 2021, compared to 2,787,561 (95%CI: 2,437,347–3,196,954) in 1990. The subsequent regions, ranked by DALYs in 2021, were East Asia (2,630,112; 95%CI: 2,122,342–3,239,598), Southeast Asia (1,302,806; 95%CI: 1,112,399–1,503,301), High-income Asia Pacific (298,552; 95%CI: 267,653–323,679), and Central Asia (105,829; 95%CI: 92,047–121,682) (Table 1, S1 Fig). Furthermore, South Asia also experienced the highest ASDR at 393.75 (95%CI: 341.05–444.26) per 100,000 population in 2021 (Table 1, Fig 1). Subsequently, Southeast Asia, East Asia, Central Asia, and High-income Asia Pacific had rates of 182.92 (95%CI: 156.58–210.59), 122.05 (95%CI: 98.8–149.31), 116.28 (95%CI: 101.45–133.20), and 73.77 (95%CI: 67.41–80.02) per 100,000 population respectively (Table 1, Fig 1). Moreover, South Asia demonstrated the highest DALY percentage across all head and neck cancers (HNC:59.65%; TC:40.76%; LC:58.28%; LOC:70.07%; OPC:78.04%), while Southeast Asia had the highest DALY percentage specifically for NPC (50.95%) in 2021 (Fig 2A, S3 Table). Additionally, LOC had the highest DALY percentage among HNC in High-income Asia Pacific (36.18%), Southeast Asia (46.23%), and Central Asia (36.09%). In contrast, NPC accounted for the highest proportion of DALYs in East Asia (38.96%). In Southeast Asia, NPC (28.93%) and LOC (32.41%) exhibited comparable contributions to DALYs (Fig 2B, S4 Table).

**Table 1:** DALYs of head and neck cancer in 1990 and 2021, and their average annual percentage changes among five GBD subregions within Asia from 1990 to 2021.

Location	Number of cases, 1990 (95%CI)	Age-standardised rate per 100,000 population, 1990 (95%CI)	Number of cases, 2021 (95%CI)	Age-standardised rate per 100,000 population, 2021 (95%CI)	Average annual percentage change, 1990–2021 (95%CI)
High-income Asia Pacific	174,797(162,853-188,090)	85.42(79.53-91.92)	298,552(267,653-323,679)	73.77(67.41-80.02)	−0.52(−0.74 to -0.30)
East Asia	2,345,727(1,979,105-2,724,800)	232.84(196.90-270.11)	2,630,112(2,122,342-3,239,598)	122.05(98.8-149.31)	−2.13(−2.36 to -1.91)
Southeast Asia	599,603(516,218-681,634)	201.93(174.50-229.48)	1,302,806(1,112,399-1,503,301)	182.92(156.58-210.59)	−0.32(−0.37 to -0.26)
Central Asia	98,244(92,173-105,408)	190.98(179.16-204.84)	105,829(92,047-121,682)	116.28(101.45-133.20)	−1.50(−1.85 to -1.14)
South Asia	2,787,561(2,437,347-3,196,954)	405.82(353.10-465.72)	6,412,639(5,537,665-7,259,268)	393.75(341.05-444.26)	−0.08(−0.17 to 0.02)

Note: CI = confidence interval.

Among 34 countries and territories, India (4,819,092; 95%CI: 4,123,094–5,494,484), China (2,453,515; 95%CI: 1,953,042–3,064,795), and Pakistan (1,055,691; 95%CI: 808,843–1,373,124) exhibited the highest DALYs in 2021 (Table 2, Fig 1). In contrast, the highest ASDRs were observed in Pakistan (695.10; 95%CI: 534.58–893.13), Seychelles (397.55; 95%CI: 327.49–472.80), and India (370.64; 95%CI: 318.42–421.72) per 100,000 population. From 1990 to 2021, Pakistan demonstrated the most significant upward trend in ASDR, with an AAPC of 0.31 (95%CI: 0.23 to 0.38) (Table 2, Fig 1). Meanwhile, Singapore experienced the most substantial decline, with an AAPC of −3.01 (95%CI: −3.15 to −2.86).

**Table 2:** DALYs of head and neck cancer in 1990 and 2021, and their average annual percentage changes among 34 countries and territories within Asia from 1990 to 2021.

Location	Number of cases, 1990 (95%CI)	Age-standardised rate per 100,000 population, 1990 (95%CI)	Number of cases, 2021 (95%CI)	Age-standardised rate per 100,000 population, 2021 (95%CI)	Average annual percentage change, 1990–2021 (95%CI)
Republic of Korea	42,042(32,602-53,869)	126.52(97.96-163.69)	57,331(44,501-73,242)	63.21(49.26-80.55)	−2.26(−2.44 to -2.07)
Japan	124,271(119,146-128,850)	73.47(70.32-76.21)	230,862(206,848-246,439)	75.720(70.39-79.80)	0.07(−0.15 to 0.29)
Taiwan (Province of China)	60,847(55,413-66,000)	335.34(306.06-363.45)	134,173(117,513-152,100)	346.68(303.92-393.11)	0.23(0.03 to 0.42)
Singapore	8,094(7,215-9,053)	301.34(267.44-338.04)	9,494(7,841-11,384)	112.56(92.62-135.21)	−3.01(−3.15 to -2.86)
Brunei Darussalam	389(308-484)	293.03(230.67-363.75)	865(698-1,061)	206.71(167.05-253.10)	−1.13(−1.32 to -0.95)
Malaysia	43,575(36,578-51,437)	384.55(321.40-454.39)	94,105(78,757-112,246)	303.35(254.29-361.99)	−0.74(−1.03 to -0.45)
Seychelles	234(197-277)	417.34(349.36-495.90)	499(410-599)	397.55(327.49-472.80)	−0.15(−0.39 to 0.09)
Kazakhstan	36,544(32,810-40,693)	261.99(234.92-291.92)	26,831(22,275-31,908)	136.74(113.55-162.47)	−2.16(−2.83 to -1.49)
Mauritius	1,384(1,296-1,487)	175.83(164.90-188.79)	2,764(2,507-2,986)	149.81(136.17-161.46)	−0.45(−1.34 to 0.43)
Georgia	13,557(11,827-15,569)	211.17(184.11-242.67)	11,851(10,129-13,734)	214.26(183.1-248.39)	−0.04(−0.57 to 0.49)
Sri Lanka	26,560(22,114-31,652)	226.75(188.87-270.30)	53,108(32,781-77,151)	194.50(120.72-280.84)	−0.44(−0.90 to 0.03)
Armenia	5,276(4,771-5,803)	174.17(157.36-191.57)	4,884(4,106-5,751)	114.82(96.34-135.49)	−1.29(−1.89 to -0.70)
Thailand	98,695(81,510-119,195)	242.34(200.39-291.15)	195,044(146,982-255,639)	188.08(142.81-244.76)	−0.82(−0.96 to -0.68)
China	2,256,922(1,890,003-2,636,208)	232.63(195.20-271.25)	2,453,515(1,953,042-3,064,795)	117.92(94.28-146.23)	−2.18(−2.45 to -1.92)
Azerbaijan	7,641(6,183-9,530)	135.97(109.85-169.04)	10,416(7,261-15,203)	89.35(62.76-129.29)	−1.31(−1.50 to -1.11)
Turkmenistan	4,888(4,372-5,404)	221.59(198.50-244.84)	7,313(5,517-9,819)	156.88(119.13-209.39)	−1.07(−1.73 to -0.40)
Indonesia	176,159(134,990-217,080)	149.54(114.22-183.86)	381,520(269,686-504,085)	142.16(101.33-185.49)	−0.16(−0.21 to -0.10)
Uzbekistan	17,685(14,372-21,638)	137.72(112.45-168.60)	30,182(23,099-38,856)	98.72(75.82-126.52)	−1.19(−1.78 to -0.61)
Philippines	60,928(51,189-71,092)	168.70(139.68-199.33)	149,211(122,405-178,838)	161.06(132.77-192.39)	−0.12(−0.2 to -0.05)
Viet Nam	120,643(91,266-157,634)	277.25(209.40-362.63)	312,577(228,871-410,884)	286.13(212.39-372.63)	0.10(0.07 to 0.13)
Mongolia	2,117(1,497-2,940)	178.45(126.80-247.11)	3,206(2,277-4,363)	114.26(81.60-154.60)	−1.42(−1.67 to -1.18)
Kyrgyzstan	7,104(5,894-8,442)	222.58(184.96-264.41)	6,267(4,834-7,966)	111.98(87.13-142.05)	−2.22(−2.58 to -1.86)
India	2,098,775(1,800,620-2,444,031)	374.49(320.05-434.74)	4,819,092(4,123,094-5,494,484)	370.64(318.42-421.72)	−0.01(−0.13 to 0.10)
Maldives	143(92-202)	145.98(100.97-198.53)	291(218-374)	76.82(58.11-98.30)	−2.18(−2.38 to -1.97)
Democratic People’s Republic of Korea	27,959(19,681-37,844)	148.21(104.79-199.02)	42,424(29,862-57,794)	124.28(88.5-168.20)	−0.57(−0.6 to -0.54)
Tajikistan	3,431(2,477-4,579)	110.52(80.46-146.97)	4,879(3,322-6,909)	67.16(46.25-94.37)	−1.63(−1.9 to -1.36)
Myanmar	53,064(35,560-74,653)	198.97(136.66-278.53)	72,780(52,110-98,510)	138.57(100.17-186.67)	−1.17(−1.25 to -1.10)
Timor-Leste	521(343-750)	142.28(95.41-201.52)	1,246(877-1,733)	135.40(95.01-188.01)	−0.15(−0.30 to 0.01)
Lao People’s Democratic Republic	5,352(3,505-7,680)	223.41(148.05-317.23)	8,866(6,151-12,163)	165.52(116.2-224.94)	−0.96(−0.99 to -0.92)
Bangladesh	245,686(177,674-313,227)	450.33(326.41-574.38)	457,226(293,334-677,841)	309.18(200.02-454.82)	−1.14(−1.32 to -0.96)
Cambodia	11,479(8,280-15,208)	216.58(158.18-285.68)	28,976(20,448-40,233)	210.31(149.47-289.88)	−0.06(−0.18 to 0.05)
Bhutan	1,100(696-1,573)	357.46(230.79-511.55)	1,907(1,290-2,768)	290.72(199.35-417.22)	−0.67(−0.75 to -0.60)
Pakistan	402,960(326,187-491,255)	635.60(516.98-774.38)	1,055,691(808,843-1,373,124)	695.10(534.58-893.13)	0.31(0.23 to 0.38)
Nepal	39,041(28,088-54,065)	348.86(250.28-479.97)	78,723(56,542-108,628)	309.91(224.63-425.90)	−0.37(−0.47 to -0.26)

Note: CI = confidence interv\al.

**Fig 3:**
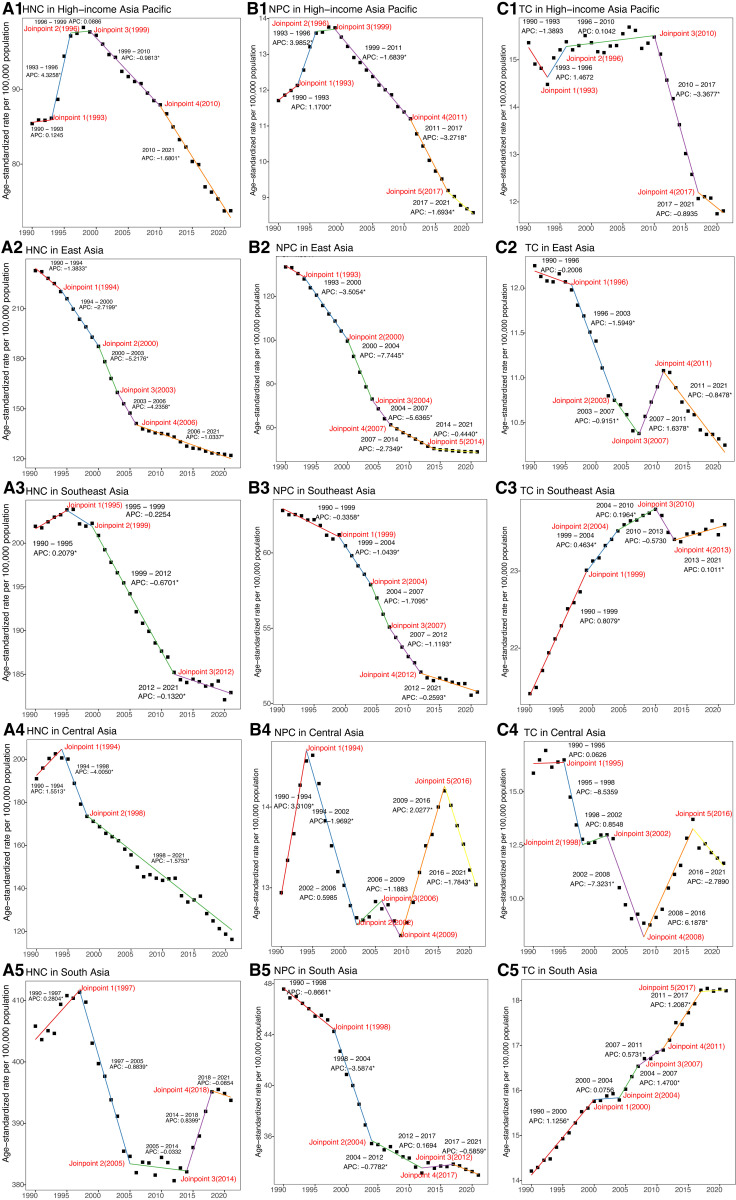
Joinpoint analysis of trends in the age-standardized DALYs rates of HNC, NPC, and TC from 1990 to 2021. (A) APCs for HNC in 5 Asia regions. (B) APCs for NPC in 5 Asia regions. (C) APCs for TC in 5 Asia regions. DALYs = Disability-Adjusted Life Years. APC = Annual percentage change. HNC = Head and neck cancer. NPC = Nasopharynx cancer. TC = Thyroid cancer. * Indicates that the APC is significantly different from zero at the alpha = 0.05 level.

The joinpoint regression results showed that there was a significant decline in ASDR across five subregions from 1990–2021, except for South Asia (AAPC = −0.08; 95%CI: −0.17 to 0.02). Notably, East Asia experienced the most rapid decrease, with an AAPC of −2.13 (95%CI: −2.36 to −1.91) (Table 1, Figs 1 and 3A). Four joinpoints were observed in the High-income Asia Pacific region, East Asia, and South Asia (Fig 3A, S5 Table). By comparison, Southeast Asia exhibited three joinpoints, and Central Asia had just two.

### Burden of nasopharynx cancer

**Fig 4:**
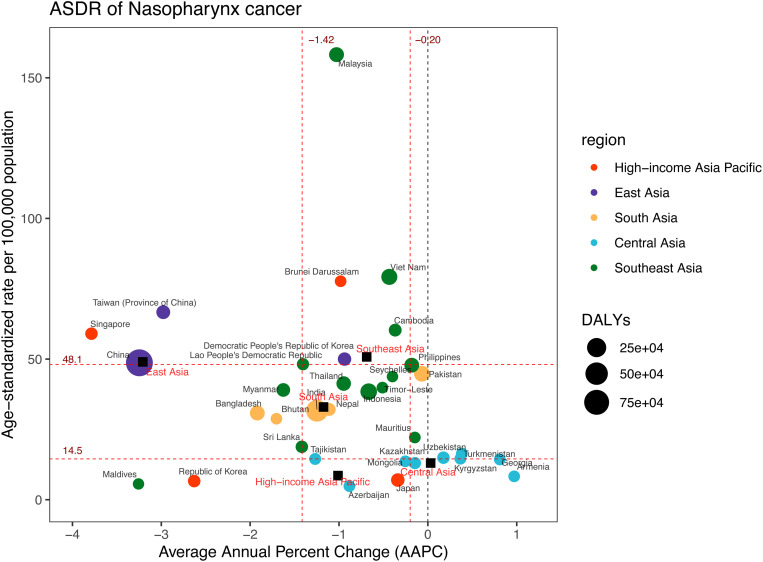
DALYs and ASDR of nasopharynx cancer in 2021, and their average annual percentage changes from 1990 to 2021. The black squares indicate the five Asia subregions. The circles’ colors identify the GBD region of each country or territory, and their sizes reflect the DALYs values. Red dashed lines indicate the tertiles of AAPC or ASDR (with corresponding values). ASDR = Age-standardised DALYs rate. DALYs = Disability-Adjusted Life Years. AAPC = Average annual percentage change.

Between 1990 and 2021, there was a rise in DALYs associated with NPC in Asia subregions except for East Asia (S6 Table, S1 Fig). In 2021, the highest DALYs were observed in East Asia, with around 1,024,646 (95%UI: 837,975–1,254,065) (Fig 2A, S6 Table, and S1 Fig). The regions that followed in descending order were South Asia 567,907 (95%UI: 495,773–640,475), Southeast Asia 376,855 (95%UI: 327,440–429,623), High-income Asia Pacific 29,562 (95%UI: 27,252–31,603), and Central Asia 12,238 (95%UI: 10,175–15,023). Southeast Asia exhibited the highest ASDR at 50.77 (95%UI: 44.30–57.72) per 100,000 population (Fig 4, S6 Table). Subsequently, the rates were 49.00 (95%UI: 40.12–59.48) per 100,000 population in East Asia, 32.96 (95%UI: 28.88–37.08) per 100,000 population in South Asia, 13.04 (95%UI: 10.86–15.98) per 100,000 population in Central Asia, and 8.58 (95%UI: 8.01–9.25) per 100,000 population in the High-income Asia Pacific region.

Across 34 countries and territories, China, India, and Indonesia were identified as having the highest DALYs in 2021, with values of 982,657 (95%UI: 797,644–1,210,379), 431,223 (95%UI: 376,460–488,651), and 110,166 (95%UI: 77,572–150,123) respectively (Fig 4, S6 Table). Conversely, Malaysia, Viet Nam, and Brunei Darussalam recorded the highest ASDR, with figures of 158.23 (95%UI: 134.38–187.14), 79.22 (95%UI: 58.34–103.27), and 77.67 (95%UI: 62.82–94.58) per 100,000 population respectively. Notably, Armenia exhibited a significant upward trend from 1990 to 2021, with an AAPC of 0.97 (95%CI: 0.40 to 1.54). In contrast, Singapore experienced the most substantial decline in ASDR over the same period, with an AAPC of −3.79 (95%CI: −4.16 to −3.41).

Between 1990 and 2021, the ASDR in Central Asia remained stable with an AAPC of 0.03 (95%CI: −0.17 to 0.24). In contrast, the other four regions experienced a declining trend in ASDR, with East Asia showing the most rapid decrease, having an AAPC of −3.21 (95%CI: −3.31 to −3.10) (S6 Table, Figs 3B and 4). In terms of joinpoints, the High-income Asia Pacific region had five, as did East Asia and Central Asia (S5 Table, Fig 3B). Southeast Asia and South Asia each featured four joinpoints.

### Burden of thyroid cancer

**Fig 5:**
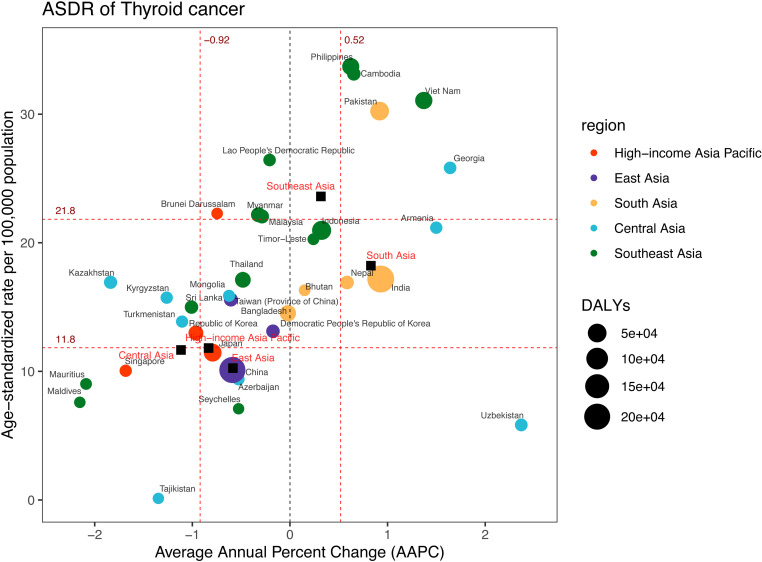
DALYs and ASDR of thyroid cancer in 2021, and their average annual percentage changes from 1990 to 2021. The black squares indicate the five Asia subregions. The black squares indicate the five Asia subregions. The circles’ colors identify the GBD region of each country or territory, and their sizes reflect the DALYs values. Red dashed lines indicate the tertiles of AAPC or ASDR (with corresponding values). ASDR = Age-standardised DALYs rate. DALYs = Disability-Adjusted Life Years. AAPC = Average annual percentage change.

From 1990 to 2021, DALYs associated with TC increased in five regions of Asia (Fig 1, S7 Table). South Asia had the highest number of DALYs in 2021, with approximately 302,257(95%UI: 249,828–356,789) (Fig 2A, S7 Table, and S1 Fig). The regions ranking second to fourth were East Asia (213,609; 95%UI: 173,066–262,362), Southeast Asia (164,547; 95%UI: 130,333–189,174), High-income Asia Pacific (50,783; 95%UI: 43,704–57,571), and Central Asia (10,273; 95%UI: 9,056–11,543). The ASDR was found to be highest in Southeast Asia, at 23.60 (95%UI: 18.80–27.01) per 100,000 population (Fig 5, S7 Table). The rates then decreased in the following order: 18.22 (95%UI: 15.09–21.35) per 100,000 population in South Asia, 11.81 (95%UI: 10.45–13.68) per 100,000 population in High-income Asia Pacific, 11.66 (95%UI: 10.33–13.03) per 100,000 population in Central Asia, and 10.25 (95%UI: 8.34–12.52) per 100,000 population in East Asia.

When examining 34 countries and territories, India, China, and Indonesia had the highest DALYs in 2021, specifically 225,288 (95%UI: 186,617–264,478), 203,325 (95%UI: 163,131–251,789), and 55,407 (95%UI: 36,366–72,736) (Fig 5, S7 Table). In the same period, Philippines, Cambodia, and Viet Nam experienced the highest ASDR, at 33.70 (95%UI: 27.26–40.74), 33.14 (95%UI: 21.33–45.39), and 31.06 (95%UI: 23.41–40.20) per 100,000 population. Uzbekistan exhibited a significant upward trend from 1990 to 2021, with an AAPC of 2.37 (95%CI: 1.79 to 2.95). By contrast, Maldives recorded the most dramatic decrease in ASDR, with an AAPC of −2.15 (95%CI: −2.38 to −1.92).

According to the joinpoint regression results, the ASDR significantly declined in High-income Asia Pacific (AAPC = −0.83; 95%CI: −1.18 to −0.49) and East Asia (AAPC = −0.58; 95%CI: −0.75 to −0.41) (Figs 3C and 5, S7 Table). However, Southeast Asia (AAPC = 0.32; 95%CI: 0.24 to 0.39) and South Asia (AAPC = 0.83; 95%CI: 0.67 to 0.99) witnessed significant growth, while Central Asia (AAPC = −1.12; 95%CI: −2.44 to 0.23) remained stable. Four joinpoints were noted in the High-income Asia Pacific region, East Asia, and Southeast Asia, while South Asia and Central Asia experienced five joinpoints each (Fig 3C, S5 Table).

### Burden of larynx cancer

**Fig 6:**
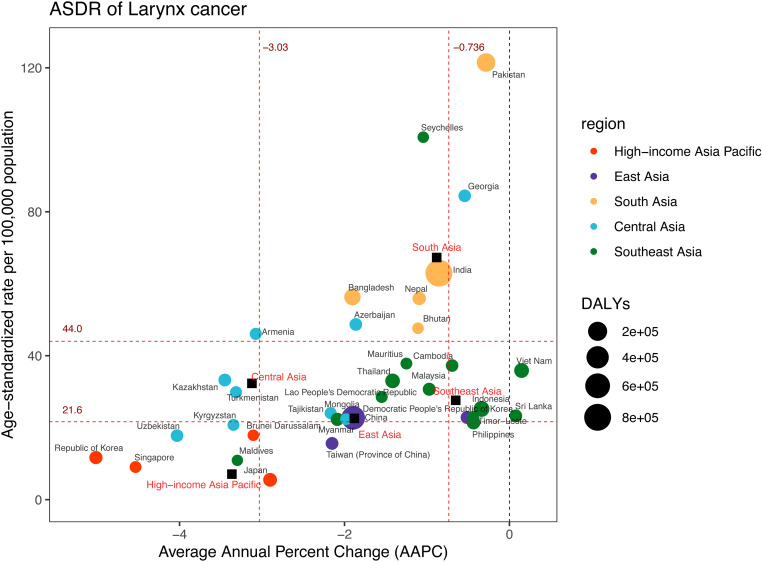
DALYs and ASDR of larynx cancer in 2021, and their average annual percentage changes from 1990 to 2021. The black squares indicate the five Asia subregions. The circles’ colors identify the GBD region of each country or territory, and their sizes reflect the DALYs values. Red dashed lines indicate the tertiles of AAPC or ASDR (with corresponding values). ASDR = Age-standardised DALYs rate. DALYs = Disability-Adjusted Life Years. AAPC = Average annual percentage change.

Between 1990 and 2021, DALYs related to LC rose in all Asia subregions outside Central Asia (S8 Table, S1 Fig). The region with the highest DALYs for LC in 2021 was South Asia, which recorded about (1,066,560; 95%UI: 929,501–1,228,037) (Fig 2A, S8 Table, and S1 Fig). Following this were East Asia (508,207; 95%UI: 397,896–639,686), Southeast Asia 194,228 (95%UI: 167,983–230,361), High-income Asia Pacific (31,591; 95%UI: 27,473–35,245), and Central Asia (29,422; 95%UI: 26,003–33,078). Regarding ASDR, South Asia was identified as having the highest ASDR, with a value of 67.29 (95%UI: 58.66–77.40) per 100,000 population (Fig 6, S8 Table). The rates in the remaining regions were Central Asia at 32.31 (95%UI: 28.65–36.25) per 100,000 population, Southeast Asia at 27.61 (95%UI: 23.90–32.69) per 100,000 population, East Asia at 22.59 (95%UI: 17.73–28.27) per 100,000 population, and the High-income Asia Pacific region at 7.10 (95%UI: 6.22–7.97) per 100,000 population.

Within 34 countries and territories, India, China, and Pakistan exhibited the highest DALYs in 2021, with respective values of 801,557 (95%UI: 690,615–926,887), 493,848 (95%UI: 382,572–626,010), and 169,489 (95%UI: 124,983–227,793) (Fig 6, S8 Table). Meanwhile, Pakistan, Seychelles, and Georgia demonstrated the highest ASDR, at 121.47 (95%UI: 88.73–161.68), 100.72 (95%UI: 81.77–121.39), and 84.43 (95%UI: 73.11–96.63) per 100,000 population. From 1990 to 2021, Viet Nam showed a considerable upward trend, with an AAPC of 0.15 (95% CI: 0.06 to 0.24). However, Republic of Korea had the most significant decline in ASDR, with an AAPC of −5.01 (95%CI: −5.27 to −4.75).

**Fig 7:**
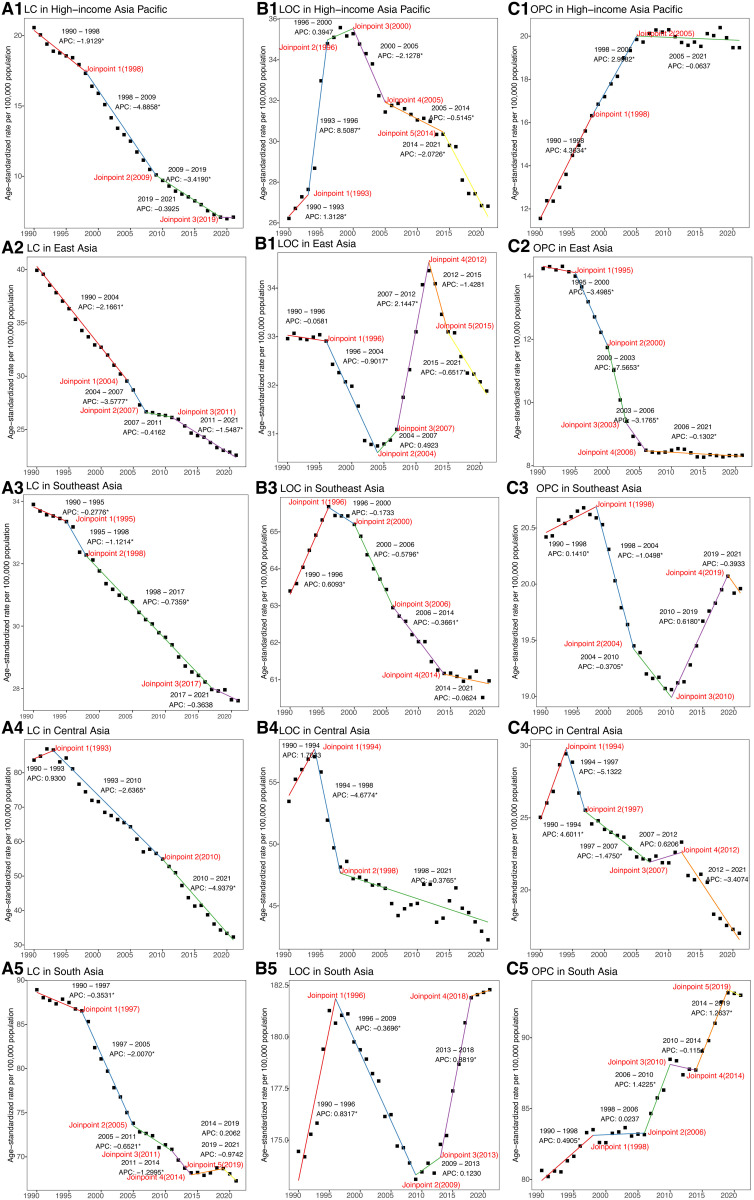
Joinpoint analysis of trends in the age-standardized DALYs rates of LC, LOC, and OPC from 1990 to 2021. (A) APCs for LC in 5 Asia regions. (B) APCs for LOC in 5 Asia regions. (C) APCs for OPC in 5 Asia regions. DALYs = Disability-Adjusted Life Years. APC = Annual percentage change. LC = Larynx cancer. LOC = Lip and oral cavity cancer. OPC = Other pharynx cancer. * Indicates that the APC is significantly different from zero at the alpha = 0.05 level.

Among the five Asian regions, the ASDR showed a significant decline, with the most pronounced decrease observed in the High-income Asia Pacific (AAPC = −3.37; 95%CI: −3.64 to −3.09) (Figs 6 and 7A, S8 Table). Three joinpoints were identified in the High-income Asia Pacific region, East Asia, and Southeast Asia (Fig 7A, S5 Table). Central Asia had two joinpoints, whereas South Asia exhibited five.

### Burden of lip and oral cavity cancer

**Fig 8:**
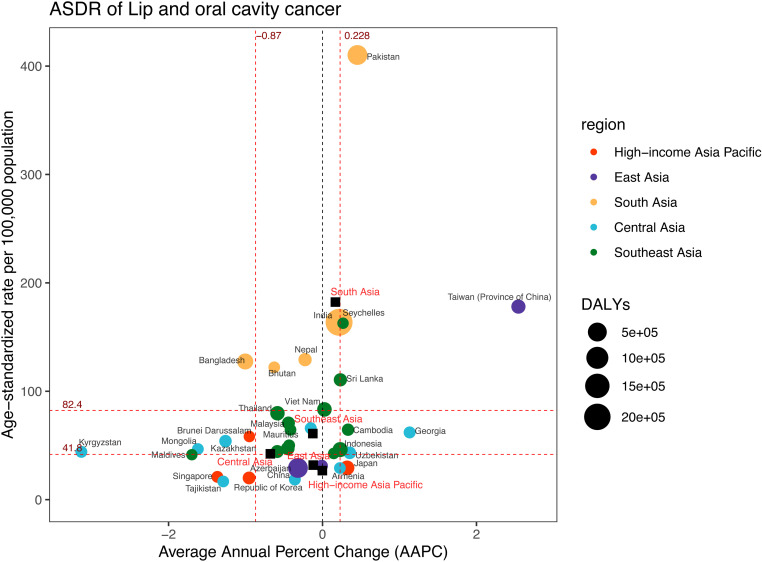
DALYs and ASDR of lip and oral cavity cancer in 2021, and their average annual percentage changes from 1990 to 2021. The black squares indicate the five Asia subregions. The circles’ colors identify the GBD region of each country or territory, and their sizes reflect the DALYs values. Red dashed lines indicate the tertiles of AAPC or ASDR (with corresponding values). ASDR = Age-standardised DALYs rate. DALYs = Disability-Adjusted Life Years. AAPC = Average annual percentage change.

During 1990–2021, an increase in LOC-related DALYs was observed across five Asian regions (S9 Table, S1 Fig). The largest DALYs were found in South Asia in 2021, amounting to around 2,964,855 (95%UI: 2,541,835–3,330,176) (Fig 2A, S9 Table, and S1 Fig). East Asia, Southeast Asia, High-income Asia Pacific, and Central Asia followed, with 697,684 (95%UI: 563,155–853,798), 422,231 (95%UI: 365,828–481,880), 108,025 (95%UI: 96,340–116,039) and 38,199 (95%UI: 33,257–43,856) respectively. South Asia exhibited the highest ASDR, which was 182.29 (95%UI: 157.06–203.68) per 100,000 population (Fig 8, S9 Table). The rates in other regions were lower, with Southeast Asia at 60.97 (95%UI: 52.95–69.50) per 100,000 population, Central Asia at 42.26 (95%UI: 36.89–48.31) per 100,000 population, East Asia at 31.88 (95%UI: 25.83–38.82) per 100,000 population, and the High-income Asia Pacific region at 26.82 (95%UI: 24.59–28.51) per 100,000 population.

An analysis of 34 countries and territories showed that India, Pakistan, and China had the highest DALYs in 2021, at 2,118,894 (95%UI: 1,793,575–2,398,412), 625,099 (95%UI: 485,940–802,124), and 618,016 (95%UI: 487,509–777,184) (Fig 8, S9 Table). Meanwhile, Pakistan, Taiwan (Province of China), and India had the highest ASDR, at 410.22 (95%UI: 321.29–518.79), 178.11 (95%UI: 160.54–197.74), and 163.61 (95%UI: 139.58–184.48) per 100,000 population. From 1990 to 2021, Taiwan (Province of China) exhibited a marked upward trajectory, with an AAPC of 2.54 (95%CI: 2.16 to 2.94). In contrast, Kyrgyzstan had the most substantial decrease in ASDR over the same period, with an AAPC of −3.13 (95%CI: −3.83 to −2.43).

The joinpoint regression findings indicated that Southeast Asia (AAPC = −0.13; 95%CI: −0.18 to −0.07) and Central Asia (AAPC = −0.68; 95%CI: −1.10 to −0.25) experienced a significant decline in ASDR (Figs 7B and 8, S9 Table). However, South Asia (AAPC = 0.17; 95%CI: 0.06 to 0.28) showed a significant rise, and High-income Asia Pacific (AAPC = 0.00; 95%CI: −0.34 to 0.34) as well as East Asia (AAPC = −0.12; 95%CI: −0.33 to 0.09) maintained stability. In the High-income Asia Pacific region and East Asia, five joinpoints were noted (Fig 7B, S5 Table). Southeast Asia and South Asia each shown four joinpoints, while Central Asia had only two.

### Burden of other pharynx cancer

**Fig 9:**
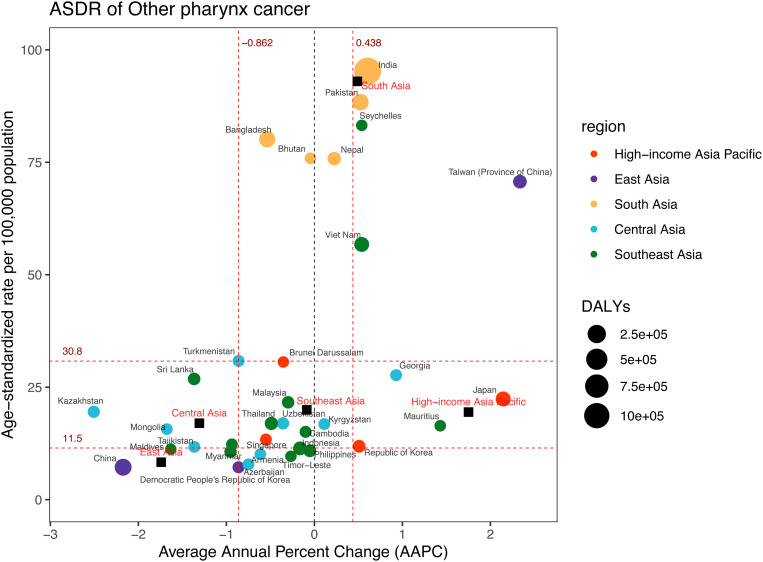
DALYs and ASDR of other pharynx cancer in 2021, and their average annual percentage changes from 1990 to 2021. The black squares indicate the five Asia subregions. The circles’ colors identify the GBD region of each country or territory, and their sizes reflect the DALYs values. Red dashed lines indicate the tertiles of AAPC or ASDR (with corresponding values). ASDR = Age-standardised DALYs rate. DALYs = Disability-Adjusted Life Years. AAPC = Average annual percentage change.

In all five regions of Asia, DALYs attributed to OPC increased between 1990 and 2021 (S10 Table, S1 Fig). South Asia was the region with the highest DALYs in 2021, at approximately 1,511,059 (95%UI: 1,320,727–1,703,791) (Fig 2A, S10 Table, and S1 Fig). Following this were East Asia 185,965 (95%UI: 150,251–229,687), Southeast Asia 144,945 (95%UI: 120,816–172,263), High-income Asia Pacific 78,591 (95%UI: 72,884–83,220), and Central Asia 15,698 (95%UI: 13,556–18,181). The ASDR was found to be the highest in South Asia, at 93.00 (95%UI: 81.35–104.74) per 100,000 population (Fig 9, S10 Table). The rates then decreased in the following order: 19.96 (95%UI: 16.63–23.67) per 100,000 population in Southeast Asia, 19.47 (95%UI: 18.14–20.61) per 100,000 population in High-income Asia Pacific, 17.00 (95%UI: 14.72–19.63) per 100,000 population in Central Asia, and 8.33 (95%UI: 6.78–10.21) per 100,000 population in East Asia.

Across 34 countries and territories, India, China, and Pakistan identified as having the largest DALYs in 2021, with respective values of 1,242,130 (95%UI: 1,075,828–1,416,056), 155,670 (95%UI: 122,186–199,433), and 130,925 (95%UI: 98,365–175,028) (Fig 9, S10 Table). In contrast, India, Pakistan, and Seychelles had the highest ASDR, with values of 95.27 (95%UI: 82.55–108.59), 88.36 (95%UI: 66.74–116.72), and 83.21 (95%UI: 66.89–100.02) per 100,000 population. Over the 1990−2021 period, Taiwan (Province of China) showed a significant upward trend, with an AAPC of 2.34 (95%CI: 1.71 to 2.97). Conversely, Kazakhstan experienced the most notable decline in ASDR, with an AAPC of −2.51 (95%CI: −3.55 to −1.45).

High-income Asia Pacific (AAPC = 1.75; 95%CI: 1.57 to 1.94) and South Asia (AAPC = 0.49; 95%CI: 0.34 to 0.63) experienced a significant rise in ASDR from 1990 to 2021 (Figs 7C and 9, S10 Table). By contrast, East Asia (AAPC = −1.74; 95%CI: −2.02 to −1.46), Southeast Asia (AAPC = −0.09; 95%CI: −0.16 to −0.01), and Central Asia (AAPC = −1.31; 95%CI: −2.00 to −0.61) exhibited notable decreases. In terms of joinpoints, the High-income Asia Pacific region had two, while East Asia, Southeast Asia, and Central Asia each shown four (Fig 7C, S5 Table). South Asia exhibited the highest number, with five joinpoints.

### Prediction from 2022 to 2030

**Fig 10:**
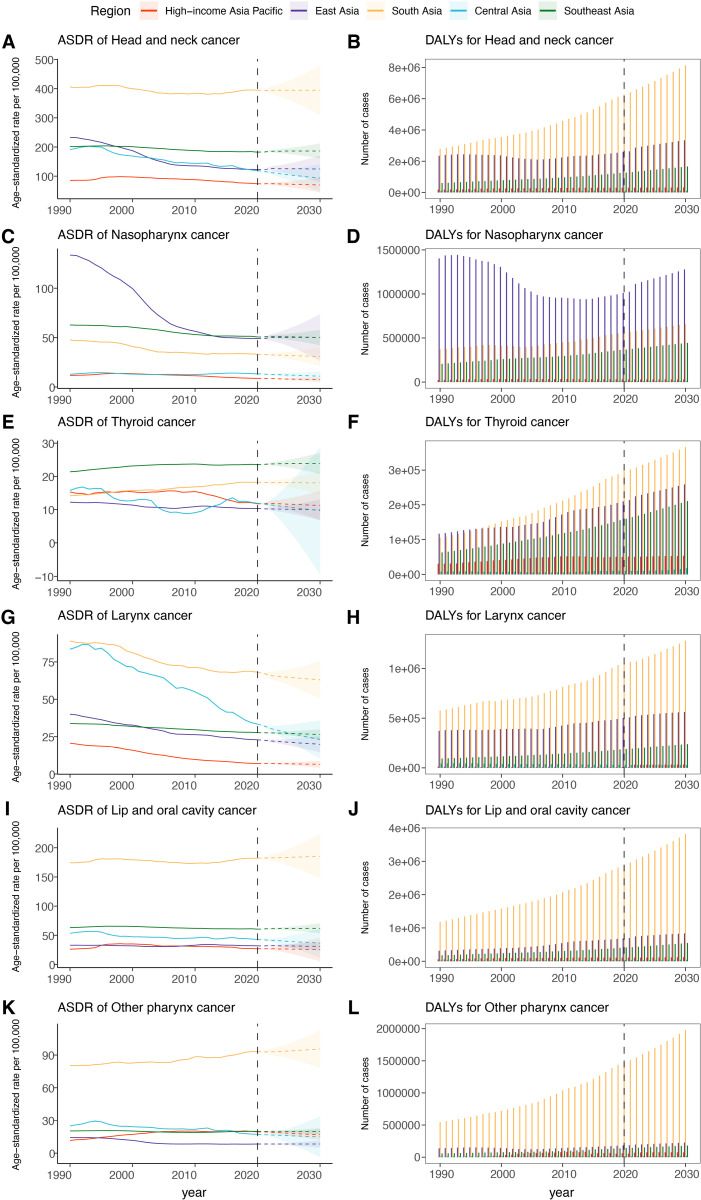
Projections of DALYs and age-standardized DALYs rates for head and neck cancers and it’s five types (nasopharynx cancer, thyroid cancer, larynx cancer, lip and oral cavity cancer, and other pharynx cancer) to 2030 at 5 Asia GBD regions. The solid line represents the observed ASDR, and the dashed line represents the ASDR predicted by the BAPC model. ASDR = Age-standardised DALYs rate. DALYs = disability adjusted life-years; BAPC = Bayesian Age-Period-Cohort.

By 2030, South Asia was projected to have the highest number and rate of DALYs for HNC (8,436,762 cases; 394.59 per 100,000 population), LC (1,346,606 cases; 62.98 per 100,000 population), LOC (3,962,212 cases; 185.31 per 100,000 population), and OPC (2,041,844 cases; 95.50 per 100,000 population) (Fig 10, S11 Table). East Asia and Southeast Asia were predicted to exhibit a similar ASDR for NPC, at around 50.00 per 100,000 population. Nevertheless, East Asia was anticipated to have the highest absolute DALYs (748,936 cases). TC was forecasted to present the highest ASDR in Southeast Asia (23.90 per 100,000 population), and the highest absolute DALYs in South Asia (385,721cases). Furthermore, LOC was expected to the most considerable disease burden in Asia regions among the five HNC subtypes.

## Discussion

This study offered the first detailed estimation of DALYs and ASDR for HNC in Asian and explored their temporal trends, with projections to 2030. Several key aspects of our findings as follows: First, significant increases in DALYs for HNC were observed in all subregions, with South Asia showing the most pronounced rise. Meanwhile, the ASDR remained stable in South Asia but declined in other regions, with the most notable decline in East Asia. Second, LOC accounted for the highest proportion of DALYs in the High-income Asia Pacific, Central Asia, and South Asia. Conversely, NPC was the leading cause of DALYs in East Asia. In Southeast Asia, NPC and LOC demonstrated similar DALY contributions. Third, South Asia exhibited the highest ASDR for LC, LOC, and OPC. Southeast Asia, however, had the highest ASDRs for NPC and TC. Fourth, at the national level, DALYs associated with HNC were predominantly concentrated in India, China, Pakistan, and Indonesia in 2021. Notably, the highest ASDRs for specific HNC subtypes were observed in the following regions: Malaysia (NPC), the Philippines (TC), Pakistan (LC, and LOC), and India (OPC). Fifth, the BAPC model projects no substantial changes in the ASDRs of HNC and its five subtypes across five Asian regions by 2030. In contrast, DALYs are projected to persistently rise. To summarize, the burden HNC across Asian regions exhibited substantial regional heterogeneity in DALYs and ASDR from 1990 to 2030.

Our investigation reveals a substantial increase in DALYs across all Asian subregions, with South Asia exhibiting the most pronounced rise. This trend aligns with the global pattern reported by Zhou et al., who documented a sustained increase in HNC-related DALYs worldwide [6]. The particularly pronounced increase in DALYs in South Asia may be explained by the significant DALYs growth observed in India and Pakistan, which were among the most notable among the 34 countries. Population growth was a key driver of the rapidly increasing burden of HNC in India and Pakistan. Between 1990 and 2021, the populations of both India and Pakistan nearly doubled. Specifically, India saw its population increase from 853,065,000 to 1,414,494,000, while the Pakistan experienced population growth from 111,127,000 to 235,553,000. Additionally, the widespread use of tobacco, including smokeless tobacco, and alcohol consumption were major attributable risk factors for HNC in India and Pakistan [15–17]. Earlier study have indicated that while the associations between alcohol and tobacco use and HNC subtypes may differ in strength, both are independently associated with overall HNC risk and show a positive multiplicative interaction [18]. Additionally, in these two countries, the percentage of adolescents who initiate smoking before age 15 is steadily increasing [19]. However, while the DALYs in South Asia have increased, the ASDR in this region exhibited a fluctuating pattern between 1990 and 2021. The ASDR peaked in 1997, declined sharply until it reached its lowest point in 2014, and then rose again. As a result, the ASDR exhibited an overall stable trend, which contrasts with the general decline in ASDRs observed across the rest of Asia subregions. The temporal trend of ASDR in South Asia could indicate a lag in advancements in the healthcare system, where late-stage diagnosis and inadequate treatment infrastructure remain major challenges [20,21]. This discrepancy suggests that while the burden of disease is increasing in South Asia, improvements in treatment efficacy may not be keeping pace with the rising incidence, which requires urgent attention [22]. In East Asia, where the most significant decline of ASDR has occurred, China has made the largest contribution. The decline could be attributed to several key factors. First, rapid economic development has led to substantial improvements in healthcare infrastructure, which has enhanced the availability and quality of medical services [23]. Second, enhanced access to early detection and advanced treatment options, including immunotherapy, radiation therapy, has played a critical role in improving survival rates of HNC [24,25]. Furthermore, the expansion of healthcare insurance policies has also promoted greater equity in healthcare access for HNC patients, particularly in rural areas, further contributing to the reduction in DALYs [26].

When breaking down the subtypes of HNC, our study revealed that the burden of specific HNC subtypes also exhibited significant regional and temporal variations. LOC accounted for the highest proportion of HNC- related DALYs in the High-income Asia Pacific, Central Asia, and South Asia in 2021. Meanwhile, NPC was the main driver of DALYs in East Asia, whereas NPC and LOC exhibited similar DALY contributions in Southeast Asia. Consistent with existing literature, lip and oral cavity cancer accounted for 389,485 incident cases (2% incidence rate) and 188,230 deaths (1.9% mortality rate) globally in 2022 [1]. These metrics were significantly higher than those for laryngeal cancer, which had 188,960 incident cases and 103,216 deaths, ranking second in the burden of head and neck cancers. The significant burden of LOC in Asian countries can be primarily attributed to traditional factors, such as poor oral hygiene, smoking, alcohol use, low fruit and vegetable intake, and the consumption of areca nut (with or without tobacco). Notably, the areca nut, ranked as the fourth most common addictive substance worldwide, has been designated as a Class I carcinogen by the International Agency for Research on Cancer (IARC) [27]. From 1990 to 2021, the most notable increase in ASDR for LOC was observed in Taiwan (Province of China), according to our research. As a previous meta-analysis reported a meta-relative risk of 10.98 (95% CI, 4.86–24.84; 13 studies) for betel quid without added tobacco (BQ-T) in Taiwan (Province of China) [28]. The risk of oral and oropharyngeal cancer increased with greater daily amounts and longer duration of betel quid chewing. If betel quid were no longer chewed, roughly half of oral cancers (the population attributable fraction% = 53.7%) in Taiwan (Province of China) could be prevented. Furthermore, human papillomavirus (HPV)-related HNC is increasingly recognized, especially in North America and Northern Europe [29]. For oral cavity cancer, HPV prevalence is higher in the Asia Pacific region (38.91%), compared to South America (33.1%), Europe (17.5%), and North America (13.4%) [30,31]. In East Asia, nasopharyngeal carcinoma (NPC) was the primary driver of DALYs in 2021, with the highest contribution from China, particularly in the southern region. Genetic and environmental factors (such as: preserved food, occupational carcinogens, and air pollutants), Epstein-Barr virus (EBV) infection, along with population size, played a crucial role in driving its high DALYs. Nevertheless, the ASDR of NPC in China had declined significantly. Such improvement was probably due to population-based screening programs, progress in imaging techniques, and the use of tailored chemoradiotherapy approaches. For example, the widespread adoption of intensity-modulated radiotherapy (IMRT) and the optimization of chemotherapy approaches (including induction, concurrent, and adjuvant therapies) minimized treatment-related toxicities while enhancing survival outcomes. In Southeast Asia, NPC and LOC demonstrated comparable contributions to DALYs. The region’s high NPC disease burden was largely attributable to Malaysia, which exhibited the highest ASDR for NPC among all 34 Asian countries and territories. The shared burden of both NPC and LOC in Southeast Asia suggests a complex interplay of risk factors, such as tobacco use and viral infections (HPV for LOC and EBV for NPC). These findings highlight the necessity for targeted public health interventions.

By employing the BAPC model, this research forecasted the disease burden of HNC and its subtypes across five Asian regions from 2021 to 2030. The projections indicated that the ASDRs for these diseases remained relatively stable, whereas the DALYs maintained an upward trend. To address the rising burden of HNC in Asia, several public health interventions need to be prioritized. First of all, a critical public health measure is the strengthening of tobacco and alcohol control programs, particularly in South Asia, where these behaviors are common. Public health campaigns focused on smoking cessation and the reduction of alcohol consumption have been successful in other regions, and similar efforts should be amplified in Asia. Given the substantial evidence linking betel nut use to an increased risk of oral cancer, targeted educational campaigns highlighting its carcinogenic effects should be implemented in high-prevalence countries/territories such as Taiwan (Province of China), India, and Pakistan. Furthermore, HPV vaccination has proven effective in reducing the incidence of cervical and other HPV-related cancers in high-income countries, and similar efforts should be prioritized in Asia. In addition to prevention, early detection is a key strategy to reduce disease burden of HNC. Screening programs for oral and nasopharyngeal cancers, especially in high-risk populations, should be scaled up [32,33]. A previous randomized controlled trial (RCT) conducted in India demonstrated a reduction in oral cancer mortality through the implementation of visual screening in high-risk populations [34]. For NPC, the detection of EBV DNA in plasma or serum has shown promise as an early diagnostic tool and could be incorporated into national screening programs, particularly in countries like China and Malaysia [33,35]. Additionally, the integration of artificial intelligence (AI) to analyze electronic health records and imaging data (e.g., CT and MRI scans, histopathological images) represents a promising strategy to extend cancer screening coverage in settings with constrained resources [36–39]. Finally, strengthening international cooperation and optimizing the allocation of healthcare resources are crucial to enhancing healthcare systems and ensuring that patients with HNC have access to timely and effective treatments, including surgery, radiotherapy, and chemotherapy. In many parts of Asia, access to radiotherapy remains limited, especially in low- and middle-income regions [40]. Expanding the availability of radiotherapy services, as well as providing financial support for cancer treatment, would help reduce DALYs from HNC.

Several limitations of this study must be acknowledged when interpreting the results. First, the analysis was heavily reliant on the 2021 GBD data, which varied in quality and availability across regions. In particular, low- and middle-income countries may have less accurate burden estimates due to limited healthcare resources. Secondly, our investigation was limited to five common HNC types: thyroid, nasopharyngeal, laryngeal, lip, and oral cancer, and other pharyngeal cancers. We did not include additional malignancies that might enhance the comprehensiveness of our findings. Furthermore, variations in diagnostic and therapeutic strategies across countries and time periods could lead to potential biases, which may undermine the comparability of results in cross-national and longitudinal comparisons. Finally, our projections to 2030, which were based on rigorous BAPC models, relied on specific assumptions that might be influenced by external factors like the COVID-19 pandemic. Future research that incorporates more recent data and employs joinpoint analysis is essential to evaluate the long-term impacts of the pandemic and validate the projections. Overall, our findings offer valuable insights into the trends of HNC in Asia, but they should be interpreted with caution, acknowledging the subtle complexities and potential biases within the broader scope of our study.

## Conclusions

In summary, our secondary analysis of the GBD 2021 database indicated that, despite a general decline in the ASDR of HNC from 1990 to 2021, the DALYs increased in all five subregions. This upward trend is projected to continue through 2030, highlighting the persistent significance of HNC as a public health challenge in Asia. Additionally, the five HNC subtypes exhibited considerable variability across the five GBD Asia subregions and 34 countries/territories. Therefore, international collaboration should be prioritized to facilitate the sharing of knowledge, research findings, and best practices among countries, enabling a unified and effective approach to HNC prevention and treatment. Policies should be tailored to local cultures and customs, while strengthening regional data monitoring to ensure that interventions target the unique epidemiological and healthcare challenges in each region, thereby reducing the disease burden and addressing health disparities in the coming decades.

## Supporting information

S1 Table List of International Classification of Diseases (ICD) codes mapped to the Global Burden of Disease cause list for head and cancers of death. (DOCX)

S2 Table Classification of 34 countries and territories into 5 Asia GBD Regions. (DOCX)

S3 Table Cancer related-DALYs percentage of five Asia GBD regions across head and neck cancers from 1990 to 2021. (DOCX)

S4 Table DALYs percentage of five head and neck cancers (nasopharynx cancer, thyroid cancer, larynx cancer, lip and oral cavity cancer, and other pharynx cancer) in five Asia GBD regions from 1990 to 2021. (DOCX)

S5 Table Joinpoint analysis of trends in age-standardized DALYs rates (ASDR) of head and neck cancers with its five types in five Asia regions, 1990–2021. (DOCX)

S6 Table DALYs of nasopharynx cancer in 1990 and 2021, and their average annual percentage changes from 1990 to 2021. (DOCX)

S7 Table DALYs of thyroid cancer in 1990 and 2021, and their average annual percentage changes from 1990 to 2021. (DOCX)

S8 Table DALYs of larynx cancer in 1990 and 2021, and their average annual percentage changes from 1990 to 2021. (DOCX)

S9 Table DALYs of lip and oral cavity cancer in 1990 and 2021, and their average annual percentage changes from 1990 to 2021. (DOCX)

S10 Table DALYs of other pharynx cancer in 1990 and 2021, and their average annual percentage changes from 1990 to 2021. (DOCX)

S11 Table Projections of DALYs and age-standardized DALYs rates for head and neck cancers and it’s five types (nasopharynx cancer, thyroid cancer, larynx cancer, lip and oral cavity cancer, and other pharynx cancer) from 2022 to 2030 at 5 Asia regions. (DOCX)

S1 Fig DALYs of head and neck cancer and its five subtypes across five Asia subregions in 1990 and 2021. (DOCX)
