# Supplementary material for: Space-time analysis of head and neck cancer in Asia and its 34 countries and territories (1990–2021): Implications from the Global Burden of Disease Study 2021
Source: PLoS One. 2025 Jun 17;20(6):e0326177. doi: 10.1371/journal.pone.0326177 (PMC12173354; doi:10.1371/journal.pone.0326177)
Supplement: S1 Table — (DOCX) [file pone.0326177.s001.docx]

# S1 Table. List of International Classification of Diseases (ICD) codes mapped to the Global Burden of Disease cause list for head and cancers of death.

| **Cause** | **ICD10** | **ICD9** |
| --- | --- | --- |
| Nasopharynx cancer | C11-C11.9, D10.6 | 147-147.9, 210.7-210.9 |
| Thyroid cancer | C73-C73.9, D09.3, D09.8, D34-D34.9, D44.0 | 193-193.9, 226-226.9 |
| Larynx cancer | C32-C32.9, D02.0, D14.1, D38.0 | 161-161.9, 212.1, 231.0, 235.6 |
| Lip and oral cavity cancer | C00-C08.9, D10.0-D10.5, D11-D11.9 | 140-145.9, 210.0-210.6, 235.0 |
| Other pharynx cancer | C09-C10.9, C12-C13.9, D10.7 | 146-146.9, 148-148.9 |
