# Supplementary material for: Space-time analysis of head and neck cancer in Asia and its 34 countries and territories (1990–2021): Implications from the Global Burden of Disease Study 2021
Source: PLoS One. 2025 Jun 17;20(6):e0326177. doi: 10.1371/journal.pone.0326177 (PMC12173354; doi:10.1371/journal.pone.0326177)
Supplement: S2 Table — (DOCX) [file pone.0326177.s002.docx]

**S2 Table.** Classification of 34 countries and territories into 5 Asia GBD Regions.

| **Region** | **34 countries and territories** |
| --- | --- |
| High - income Asia Pacific | Republic of Korea; Japan; Singapore; Brunei Darussalam. |
| East Asia | China; Taiwan (Province of China); Democratic People's Republic of Korea. |
| Southeast Asia | Malaysia; Seychelles; Mauritius; Thailand; Indonesia; Uzbekistan; Philippines; Viet Nam; Myanmar; Timor - Leste; Lao People's Democratic Republic; Cambodia; Maldives. |
| Central Asia | Kazakhstan; Georgia; Armenia; Azerbaijan; Turkmenistan; Mongolia; Kyrgyzstan; Tajikistan. |
| South Asia | India; Bangladesh; Bhutan; Pakistan; Nepal. |
