# Supplementary material for: Space-time analysis of head and neck cancer in Asia and its 34 countries and territories (1990–2021): Implications from the Global Burden of Disease Study 2021
Source: PLoS One. 2025 Jun 17;20(6):e0326177. doi: 10.1371/journal.pone.0326177 (PMC12173354; doi:10.1371/journal.pone.0326177)
Supplement: S3 Table — (DOCX) [file pone.0326177.s003.docx]

**S3 Table.** Cancer related-DALYs percentage of five Asia GBD regions across head and neck cancers from 1990 to 2021.

| **Cause** | **Year** | **High-income Asia Pacific (%)** | **East Asia (%)** | **Southeast Asia (%)** | **Central Asia (%)** | **South Asia (%)** |
| --- | --- | --- | --- | --- | --- | --- |
| Head and neck cancer | 1990 | 2.91 | 39.06 | 9.98 | 1.64 | 46.41 |
| Head and neck cancer | 1991 | 2.93 | 39.06 | 10.02 | 1.66 | 46.32 |
| Head and neck cancer | 1992 | 2.96 | 38.51 | 10.12 | 1.68 | 46.73 |
| Head and neck cancer | 1993 | 2.99 | 38.12 | 10.24 | 1.68 | 46.97 |
| Head and neck cancer | 1994 | 3.09 | 37.42 | 10.33 | 1.64 | 47.52 |
| Head and neck cancer | 1995 | 3.31 | 36.76 | 10.45 | 1.62 | 47.86 |
| Head and neck cancer | 1996 | 3.44 | 36.00 | 10.60 | 1.52 | 48.45 |
| Head and neck cancer | 1997 | 3.49 | 35.25 | 10.64 | 1.43 | 49.18 |
| Head and neck cancer | 1998 | 3.56 | 34.74 | 10.76 | 1.38 | 49.57 |
| Head and neck cancer | 1999 | 3.59 | 34.20 | 11.01 | 1.36 | 49.83 |
| Head and neck cancer | 2000 | 3.62 | 33.64 | 11.12 | 1.34 | 50.28 |
| Head and neck cancer | 2001 | 3.64 | 32.60 | 11.28 | 1.33 | 51.16 |
| Head and neck cancer | 2002 | 3.66 | 31.55 | 11.48 | 1.33 | 51.98 |
| Head and neck cancer | 2003 | 3.71 | 30.60 | 11.65 | 1.32 | 52.72 |
| Head and neck cancer | 2004 | 3.70 | 29.93 | 11.83 | 1.30 | 53.24 |
| Head and neck cancer | 2005 | 3.69 | 29.16 | 11.88 | 1.29 | 53.97 |
| Head and neck cancer | 2006 | 3.70 | 28.46 | 11.95 | 1.26 | 54.64 |
| Head and neck cancer | 2007 | 3.68 | 27.95 | 11.92 | 1.22 | 55.24 |
| Head and neck cancer | 2008 | 3.61 | 27.74 | 11.91 | 1.22 | 55.52 |
| Head and neck cancer | 2009 | 3.57 | 27.58 | 11.91 | 1.20 | 55.74 |
| Head and neck cancer | 2010 | 3.51 | 27.38 | 11.83 | 1.18 | 56.10 |
| Head and neck cancer | 2011 | 3.44 | 27.29 | 11.83 | 1.19 | 56.24 |
| Head and neck cancer | 2012 | 3.39 | 27.18 | 11.84 | 1.20 | 56.39 |
| Head and neck cancer | 2013 | 3.32 | 26.72 | 11.86 | 1.14 | 56.96 |
| Head and neck cancer | 2014 | 3.29 | 26.31 | 11.98 | 1.12 | 57.30 |
| Head and neck cancer | 2015 | 3.18 | 25.82 | 12.02 | 1.12 | 57.85 |
| Head and neck cancer | 2016 | 3.12 | 25.59 | 12.01 | 1.14 | 58.15 |
| Head and neck cancer | 2017 | 2.98 | 25.19 | 11.99 | 1.07 | 58.77 |
| Head and neck cancer | 2018 | 2.92 | 24.81 | 12.01 | 1.04 | 59.22 |
| Head and neck cancer | 2019 | 2.87 | 24.65 | 12.07 | 1.02 | 59.39 |
| Head and neck cancer | 2020 | 2.78 | 24.60 | 12.02 | 1.00 | 59.61 |
| Head and neck cancer | 2021 | 2.78 | 24.47 | 12.12 | 0.98 | 59.65 |
| Nasopharynx cancer | 1991 | 1.20 | 69.84 | 10.27 | 0.38 | 18.32 |
| Nasopharynx cancer | 1990 | 1.19 | 69.68 | 10.25 | 0.37 | 18.52 |
| Nasopharynx cancer | 1992 | 1.23 | 69.32 | 10.44 | 0.39 | 18.63 |
| Nasopharynx cancer | 1993 | 1.26 | 68.98 | 10.63 | 0.40 | 18.72 |
| Nasopharynx cancer | 1994 | 1.33 | 68.42 | 10.89 | 0.42 | 18.94 |
| Nasopharynx cancer | 1995 | 1.42 | 67.85 | 11.18 | 0.42 | 19.13 |
| Nasopharynx cancer | 1996 | 1.50 | 66.88 | 11.47 | 0.42 | 19.74 |
| Nasopharynx cancer | 1997 | 1.53 | 66.24 | 11.67 | 0.41 | 20.14 |
| Nasopharynx cancer | 1999 | 1.62 | 65.15 | 12.46 | 0.41 | 20.36 |
| Nasopharynx cancer | 1998 | 1.58 | 65.80 | 11.94 | 0.41 | 20.27 |
| Nasopharynx cancer | 2000 | 1.65 | 64.73 | 12.85 | 0.41 | 20.36 |
| Nasopharynx cancer | 2001 | 1.69 | 63.42 | 13.43 | 0.42 | 21.05 |
| Nasopharynx cancer | 2002 | 1.73 | 62.24 | 14.09 | 0.44 | 21.49 |
| Nasopharynx cancer | 2003 | 1.81 | 61.04 | 14.83 | 0.46 | 21.86 |
| Nasopharynx cancer | 2004 | 1.86 | 59.92 | 15.48 | 0.48 | 22.26 |
| Nasopharynx cancer | 2005 | 1.89 | 58.57 | 15.85 | 0.50 | 23.19 |
| Nasopharynx cancer | 2006 | 1.92 | 57.34 | 16.25 | 0.52 | 23.98 |
| Nasopharynx cancer | 2007 | 1.93 | 56.35 | 16.42 | 0.53 | 24.76 |
| Nasopharynx cancer | 2008 | 1.93 | 55.80 | 16.64 | 0.53 | 25.10 |
| Nasopharynx cancer | 2009 | 1.90 | 55.25 | 16.88 | 0.53 | 25.44 |
| Nasopharynx cancer | 2010 | 1.90 | 54.78 | 16.98 | 0.54 | 25.80 |
| Nasopharynx cancer | 2011 | 1.90 | 54.42 | 17.27 | 0.56 | 25.86 |
| Nasopharynx cancer | 2012 | 1.85 | 53.99 | 17.47 | 0.58 | 26.10 |
| Nasopharynx cancer | 2013 | 1.80 | 52.90 | 17.61 | 0.60 | 27.08 |
| Nasopharynx cancer | 2014 | 1.75 | 52.50 | 17.92 | 0.62 | 27.21 |
| Nasopharynx cancer | 2016 | 1.65 | 51.72 | 18.26 | 0.64 | 27.72 |
| Nasopharynx cancer | 2015 | 1.70 | 51.93 | 18.16 | 0.63 | 27.57 |
| Nasopharynx cancer | 2018 | 1.56 | 51.14 | 18.48 | 0.63 | 28.18 |
| Nasopharynx cancer | 2017 | 1.59 | 51.38 | 18.33 | 0.64 | 28.06 |
| Nasopharynx cancer | 2019 | 1.52 | 51.03 | 18.65 | 0.63 | 28.18 |
| Nasopharynx cancer | 2020 | 1.49 | 51.09 | 18.54 | 0.61 | 28.27 |
| Nasopharynx cancer | 2021 | 1.47 | 50.95 | 18.74 | 0.61 | 28.24 |
| Thyroid cancer | 1990 | 9.54 | 36.03 | 19.38 | 2.52 | 32.54 |
| Thyroid cancer | 1991 | 9.31 | 35.78 | 19.52 | 2.60 | 32.80 |
| Thyroid cancer | 1992 | 9.25 | 35.43 | 19.68 | 2.62 | 33.02 |
| Thyroid cancer | 1993 | 9.06 | 35.39 | 19.96 | 2.46 | 33.14 |
| Thyroid cancer | 1994 | 9.32 | 35.17 | 19.91 | 2.42 | 33.17 |
| Thyroid cancer | 1995 | 9.42 | 34.70 | 20.06 | 2.39 | 33.42 |
| Thyroid cancer | 1996 | 9.54 | 34.31 | 20.27 | 2.11 | 33.77 |
| Thyroid cancer | 1997 | 9.48 | 33.78 | 20.38 | 1.90 | 34.46 |
| Thyroid cancer | 1998 | 9.52 | 33.24 | 20.46 | 1.77 | 35.01 |
| Thyroid cancer | 1999 | 9.66 | 32.58 | 20.70 | 1.71 | 35.35 |
| Thyroid cancer | 2000 | 9.56 | 32.13 | 20.81 | 1.69 | 35.80 |
| Thyroid cancer | 2001 | 9.53 | 31.51 | 21.05 | 1.74 | 36.17 |
| Thyroid cancer | 2002 | 9.58 | 30.87 | 21.29 | 1.73 | 36.53 |
| Thyroid cancer | 2003 | 9.66 | 30.73 | 21.36 | 1.68 | 36.57 |
| Thyroid cancer | 2004 | 9.70 | 30.82 | 21.57 | 1.37 | 36.54 |
| Thyroid cancer | 2006 | 9.77 | 29.98 | 21.53 | 1.16 | 37.57 |
| Thyroid cancer | 2005 | 9.77 | 30.42 | 21.53 | 1.25 | 37.04 |
| Thyroid cancer | 2007 | 9.63 | 29.85 | 21.46 | 1.17 | 37.88 |
| Thyroid cancer | 2008 | 9.29 | 30.20 | 21.39 | 1.10 | 38.02 |
| Thyroid cancer | 2009 | 9.24 | 30.46 | 21.34 | 1.07 | 37.90 |
| Thyroid cancer | 2010 | 9.18 | 30.65 | 21.22 | 1.09 | 37.86 |
| Thyroid cancer | 2011 | 8.90 | 31.05 | 21.12 | 1.13 | 37.80 |
| Thyroid cancer | 2012 | 8.60 | 30.99 | 20.97 | 1.24 | 38.21 |
| Thyroid cancer | 2013 | 8.35 | 30.49 | 20.93 | 1.30 | 38.92 |
| Thyroid cancer | 2014 | 8.12 | 30.25 | 21.21 | 1.36 | 39.05 |
| Thyroid cancer | 2016 | 7.47 | 29.46 | 21.48 | 1.61 | 39.98 |
| Thyroid cancer | 2015 | 7.75 | 29.78 | 21.40 | 1.51 | 39.57 |
| Thyroid cancer | 2017 | 7.15 | 29.07 | 21.58 | 1.45 | 40.75 |
| Thyroid cancer | 2018 | 7.13 | 28.92 | 21.74 | 1.48 | 40.73 |
| Thyroid cancer | 2019 | 7.07 | 28.93 | 21.96 | 1.44 | 40.60 |
| Thyroid cancer | 2020 | 6.81 | 28.93 | 21.98 | 1.41 | 40.87 |
| Thyroid cancer | 2021 | 6.85 | 28.81 | 22.19 | 1.39 | 40.76 |
| Larynx cancer | 1990 | 3.76 | 33.06 | 8.31 | 3.79 | 51.09 |
| Larynx cancer | 1991 | 3.71 | 33.06 | 8.35 | 3.82 | 51.06 |
| Larynx cancer | 1992 | 3.65 | 32.54 | 8.43 | 3.90 | 51.48 |
| Larynx cancer | 1993 | 3.61 | 32.24 | 8.53 | 3.86 | 51.77 |
| Larynx cancer | 1994 | 3.62 | 31.79 | 8.59 | 3.66 | 52.34 |
| Larynx cancer | 1995 | 3.62 | 31.46 | 8.68 | 3.67 | 52.57 |
| Larynx cancer | 1996 | 3.66 | 31.03 | 8.79 | 3.52 | 53.00 |
| Larynx cancer | 1997 | 3.64 | 30.56 | 8.72 | 3.32 | 53.77 |
| Larynx cancer | 1998 | 3.58 | 30.48 | 8.85 | 3.21 | 53.87 |
| Larynx cancer | 1999 | 3.49 | 30.64 | 9.09 | 3.13 | 53.65 |
| Larynx cancer | 2000 | 3.42 | 30.77 | 9.13 | 3.10 | 53.58 |
| Larynx cancer | 2001 | 3.31 | 30.69 | 9.21 | 2.99 | 53.80 |
| Larynx cancer | 2002 | 3.19 | 30.55 | 9.38 | 2.97 | 53.91 |
| Larynx cancer | 2003 | 3.07 | 30.28 | 9.50 | 2.93 | 54.21 |
| Larynx cancer | 2004 | 3.02 | 30.26 | 9.67 | 2.90 | 54.14 |
| Larynx cancer | 2005 | 2.96 | 29.95 | 9.80 | 2.87 | 54.42 |
| Larynx cancer | 2006 | 2.82 | 29.34 | 9.93 | 2.76 | 55.15 |
| Larynx cancer | 2007 | 2.70 | 29.05 | 9.97 | 2.60 | 55.69 |
| Larynx cancer | 2008 | 2.54 | 29.06 | 9.97 | 2.61 | 55.81 |
| Larynx cancer | 2009 | 2.47 | 29.25 | 10.03 | 2.57 | 55.68 |
| Larynx cancer | 2010 | 2.36 | 29.17 | 10.00 | 2.48 | 55.98 |
| Larynx cancer | 2011 | 2.26 | 29.23 | 10.01 | 2.41 | 56.09 |
| Larynx cancer | 2012 | 2.19 | 29.49 | 10.03 | 2.34 | 55.94 |
| Larynx cancer | 2013 | 2.16 | 29.38 | 10.14 | 2.20 | 56.11 |
| Larynx cancer | 2014 | 2.13 | 29.09 | 10.28 | 2.06 | 56.44 |
| Larynx cancer | 2015 | 2.05 | 28.88 | 10.32 | 1.95 | 56.79 |
| Larynx cancer | 2016 | 1.97 | 28.83 | 10.33 | 1.98 | 56.89 |
| Larynx cancer | 2017 | 1.85 | 28.44 | 10.34 | 1.86 | 57.51 |
| Larynx cancer | 2018 | 1.77 | 27.94 | 10.38 | 1.74 | 58.16 |
| Larynx cancer | 2019 | 1.73 | 27.75 | 10.47 | 1.67 | 58.38 |
| Larynx cancer | 2020 | 1.70 | 27.79 | 10.49 | 1.64 | 58.38 |
| Larynx cancer | 2021 | 1.73 | 27.77 | 10.61 | 1.61 | 58.28 |
| Lip and oral cavity cancer | 1991 | 3.08 | 17.97 | 10.17 | 1.57 | 67.22 |
| Lip and oral cavity cancer | 1990 | 3.03 | 17.94 | 10.13 | 1.54 | 67.35 |
| Lip and oral cavity cancer | 1992 | 3.14 | 17.78 | 10.22 | 1.56 | 67.31 |
| Lip and oral cavity cancer | 1993 | 3.17 | 17.68 | 10.29 | 1.55 | 67.31 |
| Lip and oral cavity cancer | 1994 | 3.25 | 17.48 | 10.27 | 1.51 | 67.48 |
| Lip and oral cavity cancer | 1995 | 3.70 | 17.28 | 10.26 | 1.44 | 67.33 |
| Lip and oral cavity cancer | 1996 | 3.89 | 17.19 | 10.35 | 1.32 | 67.24 |
| Lip and oral cavity cancer | 1997 | 3.93 | 16.94 | 10.33 | 1.24 | 67.56 |
| Lip and oral cavity cancer | 1998 | 3.98 | 16.83 | 10.35 | 1.18 | 67.67 |
| Lip and oral cavity cancer | 1999 | 3.93 | 16.79 | 10.43 | 1.17 | 67.68 |
| Lip and oral cavity cancer | 2000 | 3.93 | 16.74 | 10.42 | 1.13 | 67.78 |
| Lip and oral cavity cancer | 2001 | 3.87 | 16.59 | 10.43 | 1.12 | 67.99 |
| Lip and oral cavity cancer | 2002 | 3.83 | 16.39 | 10.41 | 1.11 | 68.26 |
| Lip and oral cavity cancer | 2003 | 3.76 | 16.43 | 10.38 | 1.09 | 68.35 |
| Lip and oral cavity cancer | 2004 | 3.59 | 16.56 | 10.41 | 1.08 | 68.36 |
| Lip and oral cavity cancer | 2005 | 3.48 | 16.57 | 10.35 | 1.07 | 68.53 |
| Lip and oral cavity cancer | 2006 | 3.51 | 16.72 | 10.32 | 1.04 | 68.41 |
| Lip and oral cavity cancer | 2007 | 3.49 | 16.85 | 10.27 | 1.01 | 68.38 |
| Lip and oral cavity cancer | 2008 | 3.43 | 17.19 | 10.24 | 1.01 | 68.13 |
| Lip and oral cavity cancer | 2009 | 3.38 | 17.48 | 10.18 | 1.01 | 67.95 |
| Lip and oral cavity cancer | 2010 | 3.30 | 17.80 | 10.10 | 1.00 | 67.80 |
| Lip and oral cavity cancer | 2011 | 3.26 | 18.14 | 10.03 | 1.02 | 67.54 |
| Lip and oral cavity cancer | 2012 | 3.23 | 18.30 | 9.96 | 1.02 | 67.48 |
| Lip and oral cavity cancer | 2013 | 3.15 | 18.15 | 9.93 | 0.95 | 67.83 |
| Lip and oral cavity cancer | 2014 | 3.12 | 17.84 | 9.98 | 0.95 | 68.11 |
| Lip and oral cavity cancer | 2015 | 3.02 | 17.43 | 9.97 | 0.97 | 68.61 |
| Lip and oral cavity cancer | 2016 | 2.96 | 17.26 | 9.93 | 0.99 | 68.85 |
| Lip and oral cavity cancer | 2017 | 2.77 | 16.95 | 9.90 | 0.96 | 69.42 |
| Lip and oral cavity cancer | 2018 | 2.68 | 16.70 | 9.92 | 0.95 | 69.76 |
| Lip and oral cavity cancer | 2019 | 2.66 | 16.65 | 9.96 | 0.93 | 69.80 |
| Lip and oral cavity cancer | 2020 | 2.56 | 16.60 | 9.90 | 0.92 | 70.02 |
| Lip and oral cavity cancer | 2021 | 2.55 | 16.49 | 9.98 | 0.90 | 70.07 |
| Other pharynx cancer | 1991 | 3.32 | 17.62 | 7.52 | 1.69 | 69.85 |
| Other pharynx cancer | 1990 | 3.10 | 17.54 | 7.50 | 1.64 | 70.20 |
| Other pharynx cancer | 1992 | 3.32 | 17.43 | 7.57 | 1.70 | 69.98 |
| Other pharynx cancer | 1993 | 3.49 | 17.47 | 7.55 | 1.78 | 69.70 |
| Other pharynx cancer | 1994 | 3.64 | 17.20 | 7.56 | 1.79 | 69.80 |
| Other pharynx cancer | 1995 | 3.86 | 16.98 | 7.60 | 1.72 | 69.84 |
| Other pharynx cancer | 1996 | 3.96 | 16.51 | 7.61 | 1.56 | 70.35 |
| Other pharynx cancer | 1997 | 4.13 | 15.89 | 7.58 | 1.46 | 70.94 |
| Other pharynx cancer | 1998 | 4.33 | 15.35 | 7.61 | 1.38 | 71.33 |
| Other pharynx cancer | 1999 | 4.51 | 14.94 | 7.70 | 1.38 | 71.47 |
| Other pharynx cancer | 2000 | 4.60 | 14.43 | 7.69 | 1.34 | 71.95 |
| Other pharynx cancer | 2001 | 4.75 | 13.59 | 7.62 | 1.31 | 72.73 |
| Other pharynx cancer | 2002 | 4.87 | 12.61 | 7.62 | 1.30 | 73.61 |
| Other pharynx cancer | 2003 | 5.09 | 11.84 | 7.61 | 1.28 | 74.19 |
| Other pharynx cancer | 2004 | 5.22 | 11.38 | 7.62 | 1.23 | 74.55 |
| Other pharynx cancer | 2005 | 5.31 | 11.07 | 7.61 | 1.19 | 74.82 |
| Other pharynx cancer | 2006 | 5.22 | 10.90 | 7.55 | 1.18 | 75.14 |
| Other pharynx cancer | 2007 | 5.21 | 10.69 | 7.44 | 1.15 | 75.50 |
| Other pharynx cancer | 2008 | 5.15 | 10.54 | 7.38 | 1.14 | 75.79 |
| Other pharynx cancer | 2009 | 5.05 | 10.51 | 7.32 | 1.11 | 76.02 |
| Other pharynx cancer | 2010 | 4.93 | 10.37 | 7.19 | 1.08 | 76.44 |
| Other pharynx cancer | 2011 | 4.79 | 10.43 | 7.23 | 1.11 | 76.44 |
| Other pharynx cancer | 2012 | 4.74 | 10.49 | 7.31 | 1.16 | 76.31 |
| Other pharynx cancer | 2013 | 4.66 | 10.37 | 7.38 | 1.04 | 76.55 |
| Other pharynx cancer | 2014 | 4.67 | 10.21 | 7.49 | 1.03 | 76.61 |
| Other pharynx cancer | 2015 | 4.53 | 10.03 | 7.50 | 1.04 | 76.90 |
| Other pharynx cancer | 2016 | 4.54 | 10.00 | 7.50 | 1.00 | 76.96 |
| Other pharynx cancer | 2018 | 4.40 | 9.66 | 7.44 | 0.86 | 77.64 |
| Other pharynx cancer | 2017 | 4.44 | 9.86 | 7.47 | 0.88 | 77.35 |
| Other pharynx cancer | 2019 | 4.24 | 9.63 | 7.47 | 0.83 | 77.84 |
| Other pharynx cancer | 2020 | 4.09 | 9.61 | 7.45 | 0.82 | 78.03 |
| Other pharynx cancer | 2021 | 4.06 | 9.60 | 7.49 | 0.81 | 78.04 |
