# Supplementary material for: Space-time analysis of head and neck cancer in Asia and its 34 countries and territories (1990–2021): Implications from the Global Burden of Disease Study 2021
Source: PLoS One. 2025 Jun 17;20(6):e0326177. doi: 10.1371/journal.pone.0326177 (PMC12173354; doi:10.1371/journal.pone.0326177)
Supplement: S4 Table — (DOCX) [file pone.0326177.s004.docx]

**S4 Table.** DALYs percentage of five head and neck cancers (nasopharynx cancer, thyroid cancer, larynx cancer, lip and oral cavity cancer, and other pharynx cancer) in five Asia GBD regions from 1990 to 2021.

| **Location** | **Year** | **Nasopharynx cancer (%)** | **Thyroid cancer (%)** | **Larynx cancer (%)** | **Lip and oral cavity cancer (%)** | **Other pharynx cancer (%)** |
| --- | --- | --- | --- | --- | --- | --- |
| High-income Asia Pacific | 1990 | 13.70 | 17.65 | 24.24 | 30.55 | 13.85 |
| High-income Asia Pacific | 1991 | 13.70 | 17.09 | 23.57 | 30.88 | 14.77 |
| High-income Asia Pacific | 1992 | 13.76 | 17.02 | 22.89 | 31.57 | 14.76 |
| High-income Asia Pacific | 1993 | 13.79 | 16.57 | 22.29 | 31.88 | 15.47 |
| High-income Asia Pacific | 1994 | 13.79 | 16.75 | 21.57 | 32.14 | 15.74 |
| High-income Asia Pacific | 1995 | 13.55 | 15.96 | 20.10 | 34.66 | 15.73 |
| High-income Asia Pacific | 1996 | 13.47 | 15.75 | 19.48 | 35.55 | 15.75 |
| High-income Asia Pacific | 1997 | 13.36 | 15.54 | 18.96 | 35.74 | 16.40 |
| High-income Asia Pacific | 1998 | 13.32 | 15.55 | 18.23 | 35.91 | 16.99 |
| High-income Asia Pacific | 1999 | 13.33 | 15.94 | 17.43 | 35.65 | 17.65 |
| High-income Asia Pacific | 2000 | 13.06 | 15.92 | 17.03 | 35.92 | 18.07 |
| High-income Asia Pacific | 2001 | 12.87 | 15.97 | 16.40 | 35.87 | 18.90 |
| High-income Asia Pacific | 2002 | 12.65 | 16.24 | 15.70 | 35.89 | 19.52 |
| High-income Asia Pacific | 2003 | 12.51 | 16.50 | 15.01 | 35.44 | 20.54 |
| High-income Asia Pacific | 2004 | 12.49 | 16.86 | 14.83 | 34.44 | 21.37 |
| High-income Asia Pacific | 2005 | 12.34 | 17.25 | 14.47 | 33.89 | 22.06 |
| High-income Asia Pacific | 2006 | 12.13 | 17.54 | 13.71 | 34.59 | 22.02 |
| High-income Asia Pacific | 2007 | 11.98 | 17.52 | 13.13 | 34.81 | 22.56 |
| High-income Asia Pacific | 2008 | 11.94 | 17.37 | 12.61 | 35.02 | 23.06 |
| High-income Asia Pacific | 2009 | 11.68 | 17.67 | 12.31 | 35.15 | 23.19 |
| High-income Asia Pacific | 2010 | 11.58 | 17.95 | 11.94 | 35.11 | 23.43 |
| High-income Asia Pacific | 2011 | 11.51 | 17.82 | 11.63 | 35.74 | 23.30 |
| High-income Asia Pacific | 2012 | 11.24 | 17.61 | 11.42 | 36.25 | 23.47 |
| High-income Asia Pacific | 2013 | 11.03 | 17.56 | 11.39 | 36.29 | 23.73 |
| High-income Asia Pacific | 2014 | 10.69 | 17.21 | 11.30 | 36.62 | 24.18 |
| High-income Asia Pacific | 2015 | 10.58 | 16.91 | 11.20 | 36.77 | 24.55 |
| High-income Asia Pacific | 2016 | 10.37 | 16.59 | 10.89 | 36.85 | 25.30 |
| High-income Asia Pacific | 2017 | 10.37 | 16.57 | 10.69 | 36.23 | 26.14 |
| High-income Asia Pacific | 2018 | 10.22 | 16.80 | 10.42 | 35.79 | 26.77 |
| High-income Asia Pacific | 2019 | 10.02 | 16.97 | 10.32 | 36.25 | 26.43 |
| High-income Asia Pacific | 2020 | 10.06 | 16.87 | 10.45 | 36.18 | 26.44 |
| High-income Asia Pacific | 2021 | 9.90 | 17.01 | 10.58 | 36.18 | 26.32 |
| East Asia | 1990 | 59.85 | 4.97 | 15.88 | 13.46 | 5.84 |
| East Asia | 1991 | 59.85 | 4.94 | 15.79 | 13.55 | 5.88 |
| East Asia | 1992 | 59.65 | 5.00 | 15.67 | 13.73 | 5.95 |
| East Asia | 1993 | 59.29 | 5.07 | 15.63 | 13.93 | 6.08 |
| East Asia | 1994 | 58.69 | 5.22 | 15.67 | 14.27 | 6.15 |
| East Asia | 1995 | 58.19 | 5.29 | 15.71 | 14.58 | 6.22 |
| East Asia | 1996 | 57.56 | 5.41 | 15.77 | 14.99 | 6.27 |
| East Asia | 1997 | 57.23 | 5.49 | 15.79 | 15.25 | 6.25 |
| East Asia | 1998 | 56.82 | 5.56 | 15.90 | 15.56 | 6.17 |
| East Asia | 1999 | 56.13 | 5.65 | 16.08 | 16.00 | 6.15 |
| East Asia | 2000 | 55.23 | 5.76 | 16.46 | 16.46 | 6.09 |
| East Asia | 2001 | 53.97 | 5.89 | 16.97 | 17.13 | 6.03 |
| East Asia | 2002 | 52.75 | 6.08 | 17.48 | 17.82 | 5.87 |
| East Asia | 2003 | 51.19 | 6.36 | 17.91 | 18.76 | 5.78 |
| East Asia | 2004 | 49.64 | 6.62 | 18.36 | 19.63 | 5.75 |
| East Asia | 2005 | 48.38 | 6.81 | 18.56 | 20.43 | 5.83 |
| East Asia | 2006 | 47.11 | 7.00 | 18.52 | 21.40 | 5.97 |
| East Asia | 2007 | 46.08 | 7.15 | 18.59 | 22.10 | 6.09 |
| East Asia | 2008 | 44.90 | 7.35 | 18.76 | 22.84 | 6.14 |
| East Asia | 2009 | 43.82 | 7.54 | 18.87 | 23.54 | 6.23 |
| East Asia | 2010 | 42.82 | 7.68 | 18.95 | 24.23 | 6.32 |
| East Asia | 2011 | 41.68 | 7.84 | 19.00 | 25.07 | 6.40 |
| East Asia | 2012 | 40.79 | 7.92 | 19.20 | 25.61 | 6.48 |
| East Asia | 2013 | 40.20 | 7.97 | 19.25 | 26.02 | 6.56 |
| East Asia | 2014 | 40.00 | 8.01 | 19.26 | 26.13 | 6.61 |
| East Asia | 2015 | 39.78 | 8.00 | 19.41 | 26.11 | 6.70 |
| East Asia | 2016 | 39.56 | 7.98 | 19.47 | 26.19 | 6.79 |
| East Asia | 2017 | 39.52 | 7.97 | 19.42 | 26.22 | 6.87 |
| East Asia | 2018 | 39.42 | 8.03 | 19.35 | 26.28 | 6.92 |
| East Asia | 2019 | 39.18 | 8.08 | 19.32 | 26.42 | 7.00 |
| East Asia | 2020 | 39.08 | 8.10 | 19.33 | 26.47 | 7.02 |
| East Asia | 2021 | 38.96 | 8.12 | 19.32 | 26.53 | 7.07 |
| South Asia | 1990 | 13.39 | 3.78 | 20.65 | 42.52 | 19.65 |
| South Asia | 1991 | 13.24 | 3.82 | 20.56 | 42.73 | 19.65 |
| South Asia | 1992 | 13.21 | 3.84 | 20.43 | 42.84 | 19.68 |
| South Asia | 1993 | 13.06 | 3.86 | 20.37 | 43.03 | 19.68 |
| South Asia | 1994 | 12.79 | 3.88 | 20.31 | 43.36 | 19.65 |
| South Asia | 1995 | 12.60 | 3.91 | 20.17 | 43.65 | 19.66 |
| South Asia | 1996 | 12.62 | 3.96 | 20.01 | 43.56 | 19.84 |
| South Asia | 1997 | 12.47 | 4.01 | 19.91 | 43.60 | 20.00 |
| South Asia | 1998 | 12.27 | 4.10 | 19.69 | 43.84 | 20.10 |
| South Asia | 1999 | 12.04 | 4.21 | 19.32 | 44.26 | 20.18 |
| South Asia | 2000 | 11.62 | 4.29 | 19.18 | 44.58 | 20.32 |
| South Asia | 2001 | 11.41 | 4.31 | 18.96 | 44.74 | 20.57 |
| South Asia | 2002 | 11.06 | 4.36 | 18.72 | 45.05 | 20.81 |
| South Asia | 2003 | 10.64 | 4.39 | 18.62 | 45.32 | 21.03 |
| South Asia | 2004 | 10.37 | 4.41 | 18.47 | 45.56 | 21.20 |
| South Asia | 2005 | 10.35 | 4.48 | 18.22 | 45.67 | 21.28 |
| South Asia | 2006 | 10.26 | 4.57 | 18.14 | 45.59 | 21.44 |
| South Asia | 2007 | 10.24 | 4.59 | 18.03 | 45.38 | 21.75 |
| South Asia | 2008 | 10.09 | 4.62 | 18.00 | 45.23 | 22.06 |
| South Asia | 2009 | 9.98 | 4.64 | 17.78 | 45.27 | 22.32 |
| South Asia | 2010 | 9.84 | 4.63 | 17.75 | 45.06 | 22.73 |
| South Asia | 2011 | 9.61 | 4.63 | 17.69 | 45.29 | 22.77 |
| South Asia | 2012 | 9.51 | 4.71 | 17.56 | 45.51 | 22.71 |
| South Asia | 2013 | 9.65 | 4.77 | 17.24 | 45.61 | 22.72 |
| South Asia | 2014 | 9.52 | 4.75 | 17.15 | 45.80 | 22.78 |
| South Asia | 2015 | 9.43 | 4.75 | 17.03 | 45.88 | 22.92 |
| South Asia | 2016 | 9.33 | 4.77 | 16.91 | 45.98 | 23.01 |
| South Asia | 2017 | 9.25 | 4.79 | 16.83 | 46.02 | 23.10 |
| South Asia | 2018 | 9.10 | 4.74 | 16.87 | 45.98 | 23.31 |
| South Asia | 2019 | 8.98 | 4.71 | 16.87 | 45.98 | 23.47 |
| South Asia | 2020 | 8.93 | 4.72 | 16.76 | 46.08 | 23.52 |
| South Asia | 2021 | 8.86 | 4.71 | 16.63 | 46.23 | 23.56 |
| Central Asia | 1990 | 7.53 | 8.29 | 43.47 | 27.66 | 13.06 |
| Central Asia | 1991 | 7.56 | 8.42 | 42.93 | 27.86 | 13.23 |
| Central Asia | 1992 | 7.60 | 8.46 | 43.02 | 27.61 | 13.31 |
| Central Asia | 1993 | 7.87 | 7.98 | 42.37 | 27.72 | 14.06 |
| Central Asia | 1994 | 8.13 | 8.18 | 41.08 | 28.05 | 14.56 |
| Central Asia | 1995 | 8.22 | 8.28 | 41.66 | 27.53 | 14.31 |
| Central Asia | 1996 | 8.53 | 7.88 | 42.38 | 27.17 | 14.03 |
| Central Asia | 1997 | 8.74 | 7.59 | 42.14 | 27.42 | 14.12 |
| Central Asia | 1998 | 8.85 | 7.47 | 42.19 | 27.48 | 14.02 |
| Central Asia | 1999 | 8.80 | 7.46 | 41.31 | 28.12 | 14.30 |
| Central Asia | 2000 | 8.82 | 7.61 | 41.63 | 27.78 | 14.16 |
| Central Asia | 2001 | 8.81 | 7.98 | 40.55 | 28.38 | 14.28 |
| Central Asia | 2002 | 8.78 | 8.08 | 40.31 | 28.51 | 14.31 |
| Central Asia | 2003 | 8.84 | 8.05 | 40.04 | 28.65 | 14.43 |
| Central Asia | 2004 | 9.08 | 6.76 | 40.44 | 29.44 | 14.28 |
| Central Asia | 2005 | 9.36 | 6.32 | 40.34 | 29.80 | 14.18 |
| Central Asia | 2006 | 9.60 | 6.11 | 39.48 | 30.15 | 14.67 |
| Central Asia | 2007 | 9.90 | 6.44 | 38.17 | 30.40 | 15.09 |
| Central Asia | 2008 | 9.65 | 6.09 | 38.46 | 30.60 | 15.20 |
| Central Asia | 2009 | 9.57 | 6.06 | 38.11 | 31.19 | 15.07 |
| Central Asia | 2010 | 9.70 | 6.32 | 37.31 | 31.47 | 15.21 |
| Central Asia | 2011 | 9.77 | 6.51 | 35.80 | 32.27 | 15.65 |
| Central Asia | 2012 | 9.96 | 7.17 | 34.51 | 32.19 | 16.16 |
| Central Asia | 2013 | 10.74 | 8.01 | 33.89 | 31.91 | 15.44 |
| Central Asia | 2014 | 11.03 | 8.49 | 32.06 | 32.81 | 15.62 |
| Central Asia | 2015 | 11.14 | 9.30 | 30.13 | 33.53 | 15.89 |
| Central Asia | 2016 | 11.08 | 9.79 | 30.02 | 33.85 | 15.26 |
| Central Asia | 2017 | 11.54 | 9.37 | 29.91 | 34.74 | 14.44 |
| Central Asia | 2018 | 11.57 | 9.76 | 28.66 | 35.40 | 14.61 |
| Central Asia | 2019 | 11.63 | 9.71 | 28.16 | 35.85 | 14.65 |
| Central Asia | 2020 | 11.55 | 9.68 | 28.03 | 35.97 | 14.77 |
| Central Asia | 2021 | 11.56 | 9.71 | 27.80 | 36.10 | 14.83 |
| Southeast Asia | 1990 | 34.43 | 10.46 | 15.61 | 29.73 | 9.77 |
| Southeast Asia | 1991 | 34.29 | 10.50 | 15.54 | 29.88 | 9.79 |
| Southeast Asia | 1992 | 34.16 | 10.57 | 15.44 | 30.01 | 9.82 |
| Southeast Asia | 1993 | 34.02 | 10.65 | 15.38 | 30.17 | 9.78 |
| Southeast Asia | 1994 | 33.82 | 10.71 | 15.33 | 30.34 | 9.80 |
| Southeast Asia | 1995 | 33.73 | 10.76 | 15.24 | 30.47 | 9.80 |
| Southeast Asia | 1996 | 33.51 | 10.85 | 15.17 | 30.65 | 9.82 |
| Southeast Asia | 1997 | 33.41 | 10.97 | 14.93 | 30.82 | 9.87 |
| Southeast Asia | 1998 | 33.29 | 11.05 | 14.91 | 30.88 | 9.87 |
| Southeast Asia | 1999 | 33.35 | 11.15 | 14.81 | 30.86 | 9.83 |
| Southeast Asia | 2000 | 33.16 | 11.27 | 14.77 | 30.99 | 9.81 |
| Southeast Asia | 2001 | 33.01 | 11.37 | 14.72 | 31.12 | 9.78 |
| Southeast Asia | 2002 | 32.84 | 11.52 | 14.76 | 31.13 | 9.75 |
| Southeast Asia | 2003 | 32.68 | 11.62 | 14.78 | 31.16 | 9.76 |
| Southeast Asia | 2004 | 32.44 | 11.72 | 14.84 | 31.24 | 9.76 |
| Southeast Asia | 2005 | 32.12 | 11.82 | 14.91 | 31.33 | 9.82 |
| Southeast Asia | 2006 | 31.80 | 11.97 | 14.93 | 31.44 | 9.86 |
| Southeast Asia | 2007 | 31.48 | 12.05 | 14.96 | 31.58 | 9.93 |
| Southeast Asia | 2008 | 31.19 | 12.13 | 14.99 | 31.68 | 10.01 |
| Southeast Asia | 2009 | 30.99 | 12.22 | 14.99 | 31.74 | 10.06 |
| Southeast Asia | 2010 | 30.72 | 12.30 | 15.03 | 31.81 | 10.13 |
| Southeast Asia | 2011 | 30.50 | 12.31 | 15.01 | 31.95 | 10.23 |
| Southeast Asia | 2012 | 30.32 | 12.31 | 15.00 | 32.01 | 10.36 |
| Southeast Asia | 2013 | 30.14 | 12.32 | 14.96 | 32.06 | 10.52 |
| Southeast Asia | 2014 | 29.98 | 12.33 | 14.94 | 32.11 | 10.65 |
| Southeast Asia | 2015 | 29.89 | 12.36 | 14.90 | 32.08 | 10.77 |
| Southeast Asia | 2016 | 29.76 | 12.40 | 14.87 | 32.12 | 10.86 |
| Southeast Asia | 2017 | 29.62 | 12.43 | 14.84 | 32.17 | 10.94 |
| Southeast Asia | 2018 | 29.43 | 12.47 | 14.85 | 32.23 | 11.02 |
| Southeast Asia | 2019 | 29.24 | 12.52 | 14.89 | 32.27 | 11.07 |
| Southeast Asia | 2020 | 29.03 | 12.59 | 14.94 | 32.30 | 11.14 |
| Southeast Asia | 2021 | 28.93 | 12.63 | 14.91 | 32.41 | 11.13 |
