# Supplementary material for: Space-time analysis of head and neck cancer in Asia and its 34 countries and territories (1990–2021): Implications from the Global Burden of Disease Study 2021
Source: PLoS One. 2025 Jun 17;20(6):e0326177. doi: 10.1371/journal.pone.0326177 (PMC12173354; doi:10.1371/journal.pone.0326177)
Supplement: S5 Table — (DOCX) [file pone.0326177.s005.docx]

**S5 Table.** Joinpoint analysis of trends in age-standardized DALYs rates (ASDR) of head and neck cancers with its five types in five Asia regions, 1990–2021.

| **Cause** | **Location** | **Segment** | **Segment Start** | **Segment End** | **APC** | **P Value** |
| --- | --- | --- | --- | --- | --- | --- |
| Head and neck cancer | High-income Asia Pacific | 0 | 1990 | 1993 | 0.1245(-0.7938 to 1.0512) | 0.7799 |
| Head and neck cancer | High-income Asia Pacific | 1 | 1993 | 1996 | 4.3258(2.6241 to 6.0557) | <0.0001 |
| Head and neck cancer | High-income Asia Pacific | 2 | 1996 | 1999 | 0.0886(-1.3934 to 1.5929) | 0.9021 |
| Head and neck cancer | High-income Asia Pacific | 3 | 1999 | 2010 | -0.9813(-1.0958 to -0.8667) | <0.0001 |
| Head and neck cancer | High-income Asia Pacific | 4 | 2010 | 2021 | -1.6801(-1.8016 to -1.5585) | <0.0001 |
| Head and neck cancer | East Asia | 0 | 1990 | 1994 | -1.3833(-2.0826 to -0.679) | 0.0007 |
| Head and neck cancer | East Asia | 1 | 1994 | 2000 | -2.7199(-3.0964 to -2.342) | <0.0001 |
| Head and neck cancer | East Asia | 2 | 2000 | 2003 | -5.2176(-6.7048 to -3.7068) | <0.0001 |
| Head and neck cancer | East Asia | 3 | 2003 | 2006 | -4.2358(-5.6588 to -2.7913) | <0.0001 |
| Head and neck cancer | East Asia | 4 | 2006 | 2021 | -1.0337(-1.1262 to -0.9411) | <0.0001 |
| Head and neck cancer | Southeast Asia | 0 | 1990 | 1995 | 0.2079(0.05 to 0.3659) | 0.0123 |
| Head and neck cancer | Southeast Asia | 1 | 1995 | 1999 | -0.2254(-0.553 to 0.1033) | 0.1684 |
| Head and neck cancer | Southeast Asia | 2 | 1999 | 2012 | -0.6701(-0.7069 to -0.6333) | <0.0001 |
| Head and neck cancer | Southeast Asia | 3 | 2012 | 2021 | -0.132(-0.1959 to -0.0681) | 0.0003 |
| Head and neck cancer | Central Asia | 0 | 1990 | 1994 | 1.5513(0.0841 to 3.0401) | 0.0390 |
| Head and neck cancer | Central Asia | 1 | 1994 | 1998 | -4.005(-6.2863 to -1.6681) | 0.0018 |
| Head and neck cancer | Central Asia | 2 | 1998 | 2021 | -1.5753(-1.7009 to -1.4495) | <0.0001 |
| Head and neck cancer | South Asia | 0 | 1990 | 1997 | 0.2804(0.1151 to 0.446) | 0.0022 |
| Head and neck cancer | South Asia | 1 | 1997 | 2005 | -0.8839(-1.0208 to -0.7468) | <0.0001 |
| Head and neck cancer | South Asia | 2 | 2005 | 2014 | -0.0332(-0.1337 to 0.0674) | 0.4973 |
| Head and neck cancer | South Asia | 3 | 2014 | 2018 | 0.8399(0.3867 to 1.2951) | 0.0010 |
| Head and neck cancer | South Asia | 4 | 2018 | 2021 | -0.0854(-0.6466 to 0.479) | 0.7536 |
| Nasopharynx cancer | High-income Asia Pacific | 0 | 1990 | 1993 | 1.17(0.4314 to 1.9141) | 0.0041 |
| Nasopharynx cancer | High-income Asia Pacific | 1 | 1993 | 1996 | 3.9852(2.8041 to 5.1799) | <0.0001 |
| Nasopharynx cancer | High-income Asia Pacific | 2 | 1996 | 1999 | 0.1471(-0.9199 to 1.2255) | 0.7740 |
| Nasopharynx cancer | High-income Asia Pacific | 3 | 1999 | 2011 | -1.6839(-1.7625 to -1.6052) | <0.0001 |
| Nasopharynx cancer | High-income Asia Pacific | 4 | 2011 | 2017 | -3.2718(-3.5741 to -2.9685) | <0.0001 |
| Nasopharynx cancer | High-income Asia Pacific | 5 | 2017 | 2021 | -1.6934(-2.3069 to -1.0761) | <0.0001 |
| Nasopharynx cancer | East Asia | 0 | 1990 | 1993 | -1.3041(-1.8765 to -0.7283) | 0.0002 |
| Nasopharynx cancer | East Asia | 1 | 1993 | 2000 | -3.5054(-3.6446 to -3.3659) | <0.0001 |
| Nasopharynx cancer | East Asia | 2 | 2000 | 2004 | -7.7445(-8.1145 to -7.3731) | <0.0001 |
| Nasopharynx cancer | East Asia | 3 | 2004 | 2007 | -5.6365(-6.2704 to -4.9983) | <0.0001 |
| Nasopharynx cancer | East Asia | 4 | 2007 | 2014 | -2.7349(-2.87 to -2.5996) | <0.0001 |
| Nasopharynx cancer | East Asia | 5 | 2014 | 2021 | -0.444(-0.6276 to -0.26) | 0.0001 |
| Nasopharynx cancer | Southeast Asia | 0 | 1990 | 1999 | -0.3358(-0.4377 to -0.2338) | <0.0001 |
| Nasopharynx cancer | Southeast Asia | 1 | 1999 | 2004 | -1.0439(-1.3522 to -0.7345) | <0.0001 |
| Nasopharynx cancer | Southeast Asia | 2 | 2004 | 2007 | -1.7095(-2.6225 to -0.7879) | 0.0011 |
| Nasopharynx cancer | Southeast Asia | 3 | 2007 | 2012 | -1.1193(-1.4093 to -0.8285) | <0.0001 |
| Nasopharynx cancer | Southeast Asia | 4 | 2012 | 2021 | -0.2593(-0.3564 to -0.162) | <0.0001 |
| Nasopharynx cancer | Central Asia | 0 | 1990 | 1994 | 3.3109(2.8354 to 3.7887) | <0.0001 |
| Nasopharynx cancer | Central Asia | 1 | 1994 | 2002 | -1.9692(-2.2023 to -1.7356) | <0.0001 |
| Nasopharynx cancer | Central Asia | 2 | 2002 | 2006 | 0.5985(-0.2208 to 1.4246) | 0.1407 |
| Nasopharynx cancer | Central Asia | 3 | 2006 | 2009 | -1.1883(-2.756 to 0.4047) | 0.1320 |
| Nasopharynx cancer | Central Asia | 4 | 2009 | 2016 | 2.0277(1.7916 to 2.2644) | <0.0001 |
| Nasopharynx cancer | Central Asia | 5 | 2016 | 2021 | -1.7843(-2.1795 to -1.3874) | <0.0001 |
| Nasopharynx cancer | South Asia | 0 | 1990 | 1998 | -0.8661(-1.0689 to -0.6628) | <0.0001 |
| Nasopharynx cancer | South Asia | 1 | 1998 | 2004 | -3.5874(-3.9574 to -3.2159) | <0.0001 |
| Nasopharynx cancer | South Asia | 2 | 2004 | 2012 | -0.7782(-0.9847 to -0.5712) | <0.0001 |
| Nasopharynx cancer | South Asia | 3 | 2012 | 2017 | 0.1694(-0.2859 to 0.6268) | 0.4452 |
| Nasopharynx cancer | South Asia | 4 | 2017 | 2021 | -0.5859(-1.0748 to -0.0946) | 0.0221 |
| Thyroid cancer | High-income Asia Pacific | 0 | 1990 | 1993 | -1.3893(-2.9138 to 0.159) | 0.0755 |
| Thyroid cancer | High-income Asia Pacific | 1 | 1993 | 1996 | 1.4672(-1.2937 to 4.3053) | 0.2819 |
| Thyroid cancer | High-income Asia Pacific | 2 | 1996 | 2010 | 0.1042(-0.0476 to 0.2562) | 0.1664 |
| Thyroid cancer | High-income Asia Pacific | 3 | 2010 | 2017 | -3.3677(-3.9308 to -2.8013) | <0.0001 |
| Thyroid cancer | High-income Asia Pacific | 4 | 2017 | 2021 | -0.8935(-2.094 to 0.3218) | 0.1393 |
| Thyroid cancer | East Asia | 0 | 1990 | 1996 | -0.2006(-0.4915 to 0.0912) | 0.1657 |
| Thyroid cancer | East Asia | 1 | 1996 | 2003 | -1.5949(-1.8679 to -1.321) | <0.0001 |
| Thyroid cancer | East Asia | 2 | 2003 | 2007 | -0.9151(-1.712 to -0.1118) | 0.0279 |
| Thyroid cancer | East Asia | 3 | 2007 | 2011 | 1.6378(0.7692 to 2.5138) | 0.0009 |
| Thyroid cancer | East Asia | 4 | 2011 | 2021 | -0.8478(-1.0214 to -0.674) | <0.0001 |
| Thyroid cancer | Southeast Asia | 0 | 1990 | 1999 | 0.8079(0.7489 to 0.8669) | <0.0001 |
| Thyroid cancer | Southeast Asia | 1 | 1999 | 2004 | 0.4634(0.2618 to 0.6654) | 0.0001 |
| Thyroid cancer | Southeast Asia | 2 | 2004 | 2010 | 0.1964(0.0511 to 0.3418) | 0.0108 |
| Thyroid cancer | Southeast Asia | 3 | 2010 | 2013 | -0.573(-1.2444 to 0.103) | 0.0917 |
| Thyroid cancer | Southeast Asia | 4 | 2013 | 2021 | 0.1011(0.0215 to 0.1807) | 0.0156 |
| Thyroid cancer | Central Asia | 0 | 1990 | 1995 | 0.0626(-2.2971 to 2.4793) | 0.9562 |
| Thyroid cancer | Central Asia | 1 | 1995 | 1998 | -8.5359(-17.2432 to 1.0875) | 0.0767 |
| Thyroid cancer | Central Asia | 2 | 1998 | 2002 | 0.8548(-3.7929 to 5.7271) | 0.7060 |
| Thyroid cancer | Central Asia | 3 | 2002 | 2008 | -7.3231(-9.5312 to -5.061) | <0.0001 |
| Thyroid cancer | Central Asia | 4 | 2008 | 2016 | 6.1878(4.3436 to 8.0647) | <0.0001 |
| Thyroid cancer | Central Asia | 5 | 2016 | 2021 | -2.789(-5.9444 to 0.4722) | 0.0876 |
| Thyroid cancer | South Asia | 0 | 1990 | 2000 | 1.1256(1.009 to 1.2424) | <0.0001 |
| Thyroid cancer | South Asia | 1 | 2000 | 2004 | 0.0756(-0.5357 to 0.6906) | 0.7962 |
| Thyroid cancer | South Asia | 2 | 2004 | 2007 | 1.47(0.3334 to 2.6194) | 0.0146 |
| Thyroid cancer | South Asia | 3 | 2007 | 2011 | 0.5731(0.0099 to 1.1394) | 0.0465 |
| Thyroid cancer | South Asia | 4 | 2011 | 2017 | 1.2087(0.9623 to 1.4556) | <0.0001 |
| Thyroid cancer | South Asia | 5 | 2017 | 2021 | 0.0582(-0.3307 to 0.4485) | 0.7546 |
| Larynx cancer | High-income Asia Pacific | 0 | 1990 | 1998 | -1.9129(-2.3311 to -1.4929) | <0.0001 |
| Larynx cancer | High-income Asia Pacific | 1 | 1998 | 2009 | -4.8858(-5.1022 to -4.6688) | <0.0001 |
| Larynx cancer | High-income Asia Pacific | 2 | 2009 | 2019 | -3.419(-3.6676 to -3.1697) | <0.0001 |
| Larynx cancer | High-income Asia Pacific | 3 | 2019 | 2021 | -0.3925(-4.2076 to 3.5744) | 0.8361 |
| Larynx cancer | East Asia | 0 | 1990 | 2004 | -2.1661(-2.2488 to -2.0832) | <0.0001 |
| Larynx cancer | East Asia | 1 | 2004 | 2007 | -3.5777(-4.8476 to -2.2909) | <0.0001 |
| Larynx cancer | East Asia | 2 | 2007 | 2011 | -0.4162(-1.1343 to 0.3071) | 0.2441 |
| Larynx cancer | East Asia | 3 | 2011 | 2021 | -1.5487(-1.7277 to -1.3694) | <0.0001 |
| Larynx cancer | Southeast Asia | 0 | 1990 | 1995 | -0.2776(-0.5035 to -0.0512) | 0.0187 |
| Larynx cancer | Southeast Asia | 1 | 1995 | 1998 | -1.1214(-2.0529 to -0.1811) | 0.0218 |
| Larynx cancer | Southeast Asia | 2 | 1998 | 2017 | -0.7359(-0.7678 to -0.704) | <0.0001 |
| Larynx cancer | Southeast Asia | 3 | 2017 | 2021 | -0.3638(-0.733 to 0.0068) | 0.0540 |
| Larynx cancer | Central Asia | 0 | 1990 | 1993 | 0.93(-1.2986 to 3.2089) | 0.4007 |
| Larynx cancer | Central Asia | 1 | 1993 | 2010 | -2.6365(-2.8334 to -2.4391) | <0.0001 |
| Larynx cancer | Central Asia | 2 | 2010 | 2021 | -4.9379(-5.3903 to -4.4835) | <0.0001 |
| Larynx cancer | South Asia | 0 | 1990 | 1997 | -0.3531(-0.6082 to -0.0972) | 0.0101 |
| Larynx cancer | South Asia | 1 | 1997 | 2005 | -2.007(-2.2119 to -1.8017) | <0.0001 |
| Larynx cancer | South Asia | 2 | 2005 | 2011 | -0.6521(-0.9376 to -0.3658) | 0.0002 |
| Larynx cancer | South Asia | 3 | 2011 | 2014 | -1.2995(-2.5165 to -0.0673) | 0.0401 |
| Larynx cancer | South Asia | 4 | 2014 | 2019 | 0.2062(-0.177 to 0.5908) | 0.2699 |
| Larynx cancer | South Asia | 5 | 2019 | 2021 | -0.9742(-2.5471 to 0.6241) | 0.2122 |
| Lip and oral cavity cancer | High-income Asia Pacific | 0 | 1990 | 1993 | 1.3128(0.1387 to 2.5007) | 0.0307 |
| Lip and oral cavity cancer | High-income Asia Pacific | 1 | 1993 | 1996 | 8.5087(5.9747 to 11.1034) | <0.0001 |
| Lip and oral cavity cancer | High-income Asia Pacific | 2 | 1996 | 2000 | 0.3947(-0.8276 to 1.6322) | 0.5035 |
| Lip and oral cavity cancer | High-income Asia Pacific | 3 | 2000 | 2005 | -2.1278(-2.9654 to -1.283) | 0.0001 |
| Lip and oral cavity cancer | High-income Asia Pacific | 4 | 2005 | 2014 | -0.5145(-0.8555 to -0.1723) | 0.0059 |
| Lip and oral cavity cancer | High-income Asia Pacific | 5 | 2014 | 2021 | -2.0726(-2.6121 to -1.5302) | <0.0001 |
| Lip and oral cavity cancer | East Asia | 0 | 1990 | 1996 | -0.0581(-0.3277 to 0.2122) | 0.6531 |
| Lip and oral cavity cancer | East Asia | 1 | 1996 | 2004 | -0.9017(-1.0723 to -0.7309) | <0.0001 |
| Lip and oral cavity cancer | East Asia | 2 | 2004 | 2007 | 0.4923(-0.6174 to 1.6143) | 0.3608 |
| Lip and oral cavity cancer | East Asia | 3 | 2007 | 2012 | 2.1447(1.7231 to 2.5681) | <0.0001 |
| Lip and oral cavity cancer | East Asia | 4 | 2012 | 2015 | -1.4281(-2.9987 to 0.1679) | 0.0756 |
| Lip and oral cavity cancer | East Asia | 5 | 2015 | 2021 | -0.6517(-1.0518 to -0.25) | 0.0035 |
| Lip and oral cavity cancer | Southeast Asia | 0 | 1990 | 1996 | 0.6093(0.488 to 0.7306) | <0.0001 |
| Lip and oral cavity cancer | Southeast Asia | 1 | 1996 | 2000 | -0.1733(-0.505 to 0.1596) | 0.2881 |
| Lip and oral cavity cancer | Southeast Asia | 2 | 2000 | 2006 | -0.5796(-0.7221 to -0.437) | <0.0001 |
| Lip and oral cavity cancer | Southeast Asia | 3 | 2006 | 2014 | -0.3661(-0.4411 to -0.2911) | <0.0001 |
| Lip and oral cavity cancer | Southeast Asia | 4 | 2014 | 2021 | -0.0624(-0.1541 to 0.0293) | 0.1699 |
| Lip and oral cavity cancer | Central Asia | 0 | 1990 | 1994 | 1.7033(-0.1161 to 3.5558) | 0.0654 |
| Lip and oral cavity cancer | Central Asia | 1 | 1994 | 1998 | -4.6774(-7.3461 to -1.9318) | 0.0019 |
| Lip and oral cavity cancer | Central Asia | 2 | 1998 | 2021 | -0.3765(-0.5288 to -0.2238) | <0.0001 |
| Lip and oral cavity cancer | South Asia | 0 | 1990 | 1996 | 0.8317(0.6323 to 1.0315) | <0.0001 |
| Lip and oral cavity cancer | South Asia | 1 | 1996 | 2009 | -0.3696(-0.4275 to -0.3117) | <0.0001 |
| Lip and oral cavity cancer | South Asia | 2 | 2009 | 2013 | 0.123(-0.4029 to 0.6516) | 0.6298 |
| Lip and oral cavity cancer | South Asia | 3 | 2013 | 2018 | 0.8819(0.5468 to 1.2181) | <0.0001 |
| Lip and oral cavity cancer | South Asia | 4 | 2018 | 2021 | 0.0504(-0.6576 to 0.7635) | 0.8831 |
| Other pharynx cancer | High-income Asia Pacific | 0 | 1990 | 1998 | 4.3634(3.9736 to 4.7547) | <0.0001 |
| Other pharynx cancer | High-income Asia Pacific | 1 | 1998 | 2005 | 2.9982(2.3795 to 3.6206) | <0.0001 |
| Other pharynx cancer | High-income Asia Pacific | 2 | 2005 | 2021 | -0.0637(-0.2456 to 0.1185) | 0.4771 |
| Other pharynx cancer | East Asia | 0 | 1990 | 1995 | -0.2793(-0.9442 to 0.3901) | 0.3914 |
| Other pharynx cancer | East Asia | 1 | 1995 | 2000 | -3.4985(-4.1868 to -2.8052) | <0.0001 |
| Other pharynx cancer | East Asia | 2 | 2000 | 2003 | -7.5653(-9.3564 to -5.7389) | <0.0001 |
| Other pharynx cancer | East Asia | 3 | 2003 | 2006 | -3.1765(-4.8693 to -1.4536) | 0.0012 |
| Other pharynx cancer | East Asia | 4 | 2006 | 2021 | -0.1302(-0.2351 to -0.0252) | 0.0179 |
| Other pharynx cancer | Southeast Asia | 0 | 1990 | 1998 | 0.141(0.051 to 0.231) | 0.0040 |
| Other pharynx cancer | Southeast Asia | 1 | 1998 | 2004 | -1.0498(-1.2112 to -0.8881) | <0.0001 |
| Other pharynx cancer | Southeast Asia | 2 | 2004 | 2010 | -0.3705(-0.5335 to -0.2072) | 0.0002 |
| Other pharynx cancer | Southeast Asia | 3 | 2010 | 2019 | 0.618(0.5334 to 0.7027) | <0.0001 |
| Other pharynx cancer | Southeast Asia | 4 | 2019 | 2021 | -0.3933(-1.3593 to 0.5822) | 0.4067 |
| Other pharynx cancer | Central Asia | 0 | 1990 | 1994 | 4.6011(2.4877 to 6.7582) | 0.0002 |
| Other pharynx cancer | Central Asia | 1 | 1994 | 1997 | -5.1322(-10.4063 to 0.4523) | 0.0688 |
| Other pharynx cancer | Central Asia | 2 | 1997 | 2007 | -1.475(-2.0227 to -0.9243) | <0.0001 |
| Other pharynx cancer | Central Asia | 3 | 2007 | 2012 | 0.6206(-1.4757 to 2.7616) | 0.5447 |
| Other pharynx cancer | Central Asia | 4 | 2012 | 2021 | -3.4074(-4.0903 to -2.7197) | <0.0001 |
| Other pharynx cancer | South Asia | 0 | 1990 | 1998 | 0.4905(0.304 to 0.6774) | <0.0001 |
| Other pharynx cancer | South Asia | 1 | 1998 | 2006 | 0.0237(-0.1682 to 0.216) | 0.7958 |
| Other pharynx cancer | South Asia | 2 | 2006 | 2010 | 1.4225(0.8093 to 2.0394) | 0.0002 |
| Other pharynx cancer | South Asia | 3 | 2010 | 2014 | -0.1154(-0.644 to 0.416) | 0.6494 |
| Other pharynx cancer | South Asia | 4 | 2014 | 2019 | 1.2637(0.9564 to 1.5719) | <0.0001 |
| Other pharynx cancer | South Asia | 5 | 2019 | 2021 | -0.2308(-1.4782 to 1.0324) | 0.7010 |

APC = Annual percentage change.
