# Supplementary material for: Space-time analysis of head and neck cancer in Asia and its 34 countries and territories (1990–2021): Implications from the Global Burden of Disease Study 2021
Source: PLoS One. 2025 Jun 17;20(6):e0326177. doi: 10.1371/journal.pone.0326177 (PMC12173354; doi:10.1371/journal.pone.0326177)
Supplement: S6 Table — (DOCX) [file pone.0326177.s006.docx]

**S6 Table.** DALYs of nasopharynx cancer in 1990 and 2021, and their average annual percentage changes from 1990 to 2021.

| **Location** | **Number of DALYs in 1990 (95%UI)** | **Number of DALYs in 2021 (95%UI)** | **ASDR in 1990 (per 100,000 population,95%UI)** | **ASDR in 2021 (per 100,000 population, 95%UI)** | **AAPC of ASDR (95%CI)** |
| --- | --- | --- | --- | --- | --- |
| High-income Asia Pacific | 23956(22710 - 25156) | 29562(27252 - 31603) | 11.71(11.1 - 12.3) | 8.58(8.01 - 9.25) | -1.01 (-1.19 to -0.83) |
| East Asia | 1403841(1194223 - 1621286) | 1024646(837975 - 1254065) | 133.45(113.61 - 154.1) | 49(40.12 - 59.48) | -3.21 (-3.31 to -3.1) |
| Southeast Asia | 206433(180099 - 235782) | 376855(327440 - 429623) | 62.81(54.84 - 71.85) | 50.77(44.3 - 57.72) | -0.69 (-0.8 to -0.58) |
| Central Asia | 7394(6539 - 8432) | 12238(10175 - 15023) | 12.94(11.47 - 14.65) | 13.04(10.86 - 15.98) | 0.03 (-0.17 to 0.24) |
| South Asia | 373203(321842 - 430997) | 567907(495773 - 640475) | 47.58(41.01 - 55.07) | 32.96(28.88 - 37.08) | -1.17 (-1.31 to -1.04) |
| Republic of Korea | 5676(4692 - 6588) | 5645(4774 - 6544) | 15.06(12.45 - 17.44) | 6.63(5.61 - 7.67) | -2.63 (-2.79 to -2.47) |
| Japan | 12539(12101 - 12967) | 18684(17223 - 19866) | 7.57(7.31 - 7.83) | 7(6.61 - 7.4) | -0.34 (-0.64 to -0.04) |
| Taiwan (Province of China) | 31551(28408 - 34427) | 24948(19941 - 30799) | 168.83(152.48 - 184.22) | 66.68(53.22 - 82.5) | -2.98 (-3.35 to -2.6) |
| Singapore | 5575(4967 - 6259) | 4879(3777 - 6101) | 195.38(173.1 - 220.53) | 59.03(45.54 - 73.99) | -3.79 (-4.16 to -3.41) |
| Brunei Darussalam | 166(133 - 202) | 354(287 - 431) | 106.19(84.08 - 128.82) | 77.67(62.82 - 94.58) | -0.98 (-1.2 to -0.77) |
| Malaysia | 26831(22846 - 31545) | 50755(42963 - 60013) | 219.48(186.09 - 256.92) | 158.23(134.38 - 187.14) | -1.03 (-1.31 to -0.74) |
| Seychelles | 29(24 - 34) | 56(46 - 68) | 50.76(42.54 - 59.8) | 43.78(35.82 - 53.08) | -0.4 (-0.6 to -0.2) |
| Kazakhstan | 2071(1849 - 2473) | 2550(2023 - 3188) | 14.01(12.48 - 16.7) | 13.02(10.35 - 16.28) | -0.15 (-0.6 to 0.31) |
| Mauritius | 202(184 - 223) | 392(341 - 443) | 23.57(21.48 - 26) | 22.12(19.25 - 25) | -0.15 (-0.82 to 0.53) |
| Georgia | 682(508 - 899) | 702(548 - 885) | 11.21(8.35 - 14.74) | 14.31(10.99 - 17.98) | 0.81 (-0.13 to 1.76) |
| Sri Lanka | 4062(3394 - 4918) | 5049(3271 - 7246) | 30.76(25.58 - 36.9) | 18.8(12.21 - 26.75) | -1.42 (-1.83 to -1.01) |
| Armenia | 200(161 - 239) | 323(262 - 389) | 6.37(5.19 - 7.6) | 8.31(6.73 - 10.04) | 0.97 (0.4 to 1.54) |
| Thailand | 25397(21509 - 30029) | 40553(30858 - 51858) | 55.67(47.06 - 64.93) | 41.3(31.9 - 52.36) | -0.95 (-1.1 to -0.79) |
| China | 1359140(1149643 - 1576295) | 982657(797644 - 1210379) | 134.12(113.49 - 155.5) | 48.67(39.59 - 59.48) | -3.25 (-3.35 to -3.14) |
| Azerbaijan | 398(267 - 578) | 549(356 - 801) | 6.32(4.39 - 8.97) | 4.84(3.19 - 7.04) | -0.88 (-1.31 to -0.45) |
| Turkmenistan | 407(350 - 465) | 807(610 - 1086) | 14.96(13.02 - 16.93) | 16.4(12.47 - 21.84) | 0.38 (-0.36 to 1.13) |
| Indonesia | 62125(49206 - 77402) | 110166(77572 - 150123) | 47.4(37.53 - 59.02) | 38.4(27.46 - 51.32) | -0.67 (-0.73 to -0.6) |
| Uzbekistan | 2192(1534 - 3107) | 4812(3380 - 6776) | 14.14(9.93 - 19.98) | 14.94(10.5 - 20.99) | 0.18 (-0.2 to 0.56) |
| Philippines | 20634(18465 - 23369) | 47336(39184 - 56739) | 50.76(44.7 - 58.32) | 47.83(39.66 - 57.22) | -0.18 (-0.24 to -0.13) |
| Viet Nam | 41926(31907 - 54596) | 88504(64537 - 116712) | 90.99(68.52 - 118.21) | 79.22(58.34 - 103.27) | -0.43 (-0.51 to -0.36) |
| Mongolia | 216(135 - 337) | 416(265 - 597) | 14.57(9.29 - 22.71) | 13.64(8.72 - 19.56) | -0.25 (-0.6 to 0.09) |
| Kyrgyzstan | 443(335 - 588) | 884(621 - 1205) | 13.18(9.92 - 17.76) | 14.59(10.23 - 19.96) | 0.37 (0.19 to 0.54) |
| India | 295498(251035 - 341652) | 431223(376460 - 488651) | 46.74(39.72 - 54.23) | 31.62(27.68 - 35.8) | -1.25 (-1.41 to -1.08) |
| Maldives | 18(11 - 24) | 26(20 - 33) | 15.18(9.62 - 19.81) | 5.61(4.33 - 7.22) | -3.26 (-3.45 to -3.06) |
| Democratic People's Republic of Korea | 13150(9169 - 17574) | 17042(12328 - 22964) | 66.9(46.54 - 88.95) | 50(36.48 - 67.01) | -0.94 (-0.98 to -0.89) |
| Tajikistan | 786(546 - 1099) | 1194(818 - 1747) | 21.5(15.25 - 30.13) | 14.48(9.73 - 21.34) | -1.27 (-1.56 to -0.98) |
| Myanmar | 18715(13244 - 24458) | 21398(15943 - 28155) | 64.39(46.59 - 84.28) | 39.02(29.34 - 50.74) | -1.63 (-1.72 to -1.53) |
| Timor-Leste | 200(132 - 302) | 386(275 - 541) | 46.16(30.64 - 69.44) | 39.85(28.31 - 55.68) | -0.51 (-0.87 to -0.16) |
| Lao People's Democratic Republic | 1948(1243 - 2682) | 2840(2000 - 3905) | 74.72(48.02 - 101.77) | 48.17(34.42 - 65.56) | -1.41 (-1.45 to -1.36) |
| Bangladesh | 37674(26915 - 47400) | 47898(30340 - 71857) | 57.01(41.28 - 71.38) | 30.8(19.67 - 46.12) | -1.92 (-2.17 to -1.67) |
| Cambodia | 4045(3026 - 5109) | 8868(6484 - 11849) | 68.35(51.27 - 86.54) | 60.29(44.06 - 80.88) | -0.37 (-0.46 to -0.27) |
| Bhutan | 181(116 - 261) | 202(126 - 323) | 48.77(31.96 - 69.83) | 28.78(18.31 - 45.92) | -1.7 (-1.85 to -1.56) |
| Pakistan | 33886(28459 - 40754) | 79876(63047 - 100968) | 46.23(38.9 - 56.05) | 44.8(35.26 - 56.36) | -0.07 (-0.17 to 0.03) |
| Nepal | 5965(4327 - 8086) | 8708(6196 - 12044) | 45.56(33.19 - 61.74) | 32.09(22.95 - 44.24) | -1.11 (-1.21 to -1.01) |

DALYs = Disability-Adjusted Life Years. ASDR = Age-standardised DALYs rate. AAPC = Average annual percentage change. UI, Uncertainty Interval. CI, confidence interval.
