# Supplementary material for: Space-time analysis of head and neck cancer in Asia and its 34 countries and territories (1990–2021): Implications from the Global Burden of Disease Study 2021
Source: PLoS One. 2025 Jun 17;20(6):e0326177. doi: 10.1371/journal.pone.0326177 (PMC12173354; doi:10.1371/journal.pone.0326177)
Supplement: S7 Table — (DOCX) [file pone.0326177.s007.docx]

**S7 Table.** DALYs of thyroid cancer in 1990 and 2021, and their average annual percentage changes from 1990 to 2021.

| **Location** | **Number of DALYs in 1990 (95%UI)** | **Number of DALYs in 2021 (95%UI)** | **ASDR in 1990 (per 100,000 population,95%UI)** | **ASDR in 2021 (per 100,000 population, 95%UI)** | **AAPC of ASDR (95%CI)** |
| --- | --- | --- | --- | --- | --- |
| High-income Asia Pacific | 30856(28505 - 35131) | 50783(43704 - 57571) | 15.36(14.15 - 17.48) | 11.81(10.45 - 13.68) | -0.83 (-1.18 to -0.49) |
| East Asia | 116582(98062 - 136721) | 213609(173066 - 262362) | 12.25(10.38 - 14.2) | 10.25(8.34 - 12.52) | -0.58 (-0.75 to -0.41) |
| Southeast Asia | 62695(51465 - 70375) | 164547(130333 - 189174) | 21.41(17.91 - 24.29) | 23.6(18.8 - 27.01) | 0.32 (0.24 to 0.39) |
| Central Asia | 8145(7568 - 8817) | 10273(9056 - 11543) | 15.87(14.74 - 17.22) | 11.66(10.33 - 13.03) | -1.12 (-2.44 to 0.23) |
| South Asia | 105303(88400 - 135793) | 302257(249828 - 356789) | 14.21(11.98 - 18.18) | 18.22(15.09 - 21.35) | 0.83 (0.67 to 0.99) |
| Republic of Korea | 5529(4125 - 9409) | 11532(9175 - 16858) | 17.28(12.86 - 30.63) | 12.98(10.35 - 18.94) | -0.96 (-1.26 to -0.66) |
| Japan | 24889(23406 - 26351) | 38321(32740 - 42214) | 14.82(13.9 - 15.7) | 11.45(10.21 - 12.54) | -0.79 (-1.26 to -0.32) |
| Taiwan (Province of China) | 3396(3118 - 3701) | 5934(5142 - 6741) | 19.31(17.76 - 20.84) | 15.58(13.54 - 17.79) | -0.61 (-1 to -0.21) |
| Singapore | 406(347 - 467) | 848(708 - 1036) | 17.13(14.58 - 19.76) | 10.05(8.38 - 12.27) | -1.68 (-2.02 to -1.34) |
| Brunei Darussalam | 32(24 - 43) | 82(64 - 104) | 27.92(20.84 - 36.49) | 22.28(17.38 - 28.15) | -0.75 (-0.89 to -0.6) |
| Malaysia | 2437(2029 - 2867) | 6609(5601 - 8034) | 22.88(19.13 - 28.03) | 22.05(18.64 - 27.06) | -0.29 (-0.4 to -0.17) |
| Seychelles | 5(4 - 6) | 9(7 - 10) | 8.31(6.85 - 10.11) | 7.09(5.76 - 8.48) | -0.53 (-0.71 to -0.35) |
| Kazakhstan | 4486(4001 - 5042) | 3222(2644 - 3826) | 32.33(28.69 - 36.47) | 16.93(13.88 - 20.07) | -1.84 (-3.81 to 0.17) |
| Mauritius | 119(111 - 127) | 166(150 - 181) | 15.34(14.38 - 16.42) | 9.02(8.2 - 9.82) | -2.09 (-4.36 to 0.24) |
| Georgia | 910(749 - 1100) | 1404(1152 - 1700) | 14.6(11.98 - 17.7) | 25.82(21.3 - 31.46) | 1.64 (-0.04 to 3.35) |
| Sri Lanka | 2479(1922 - 2922) | 4044(2524 - 5800) | 20.98(16.07 - 24.73) | 15(9.42 - 21.37) | -1.01 (-1.34 to -0.67) |
| Armenia | 397(300 - 510) | 892(676 - 1144) | 13.69(10.35 - 17.5) | 21.17(16 - 27.26) | 1.5 (0.7 to 2.31) |
| Thailand | 7828(6437 - 10637) | 17720(13111 - 26321) | 19.78(16.14 - 27.11) | 17.12(12.66 - 25.43) | -0.48 (-0.66 to -0.31) |
| China | 110736(92143 - 130509) | 203325(163131 - 251789) | 12.09(10.14 - 14.08) | 10.1(8.14 - 12.45) | -0.59 (-0.79 to -0.39) |
| Azerbaijan | 611(462 - 826) | 1056(768 - 1421) | 11.01(8.34 - 14.68) | 9.4(6.92 - 12.41) | -0.53 (-0.86 to -0.19) |
| Turkmenistan | 433(391 - 479) | 638(488 - 832) | 19.08(17.28 - 21.12) | 13.87(10.72 - 17.88) | -1.1 (-3.16 to 1) |
| Indonesia | 21968(15720 - 26123) | 55407(36366 - 72736) | 19.02(13.94 - 22.43) | 20.96(14.04 - 27.27) | 0.32 (0.27 to 0.38) |
| Uzbekistan | 364(275 - 478) | 1750(1291 - 2371) | 2.79(2.12 - 3.67) | 5.83(4.33 - 7.83) | 2.37 (1.79 to 2.95) |
| Philippines | 9966(7534 - 12203) | 30428(24389 - 36937) | 28.17(21.39 - 34.97) | 33.7(27.26 - 40.74) | 0.62 (0.52 to 0.72) |
| Viet Nam | 9109(6839 - 12595) | 32388(23961 - 42160) | 20.48(15.17 - 29.5) | 31.06(23.41 - 40.2) | 1.37 (1.29 to 1.45) |
| Mongolia | 233(173 - 328) | 436(334 - 576) | 19.13(14.25 - 26.58) | 15.88(12.15 - 20.89) | -0.62 (-1.02 to -0.22) |
| Kyrgyzstan | 706(553 - 885) | 866(655 - 1131) | 21.45(16.85 - 26.79) | 15.73(12.03 - 20.42) | -1.26 (-3.14 to 0.65) |
| India | 77650(64371 - 102706) | 225288(186617 - 264478) | 13.04(10.89 - 17.06) | 17.18(14.23 - 20.03) | 0.93 (0.77 to 1.09) |
| Maldives | 15(9 - 20) | 29(22 - 37) | 14.47(9.01 - 19.23) | 7.59(5.73 - 9.68) | -2.15 (-2.38 to -1.92) |
| Democratic People's Republic of Korea | 2450(1722 - 3326) | 4350(3110 - 6010) | 13.86(9.89 - 18.74) | 13.12(9.47 - 18.11) | -0.17 (-0.2 to -0.15) |
| Tajikistan | 6(4 - 8) | 9(6 - 12) | 0.19(0.13 - 0.27) | 0.12(0.09 - 0.17) | -1.35 (-1.71 to -0.99) |
| Myanmar | 6520(4318 - 8598) | 11496(7886 - 15296) | 24.48(16.76 - 32.08) | 22.18(15.42 - 29.46) | -0.32 (-0.38 to -0.27) |
| Timor-Leste | 65(40 - 92) | 186(120 - 266) | 18.59(11.94 - 26.1) | 20.27(13 - 29.18) | 0.24 (0.11 to 0.37) |
| Lao People's Democratic Republic | 665(399 - 961) | 1378(889 - 1922) | 28.18(17.06 - 40.18) | 26.43(17.28 - 36.29) | -0.21 (-0.26 to -0.15) |
| Bangladesh | 9753(7403 - 13331) | 22092(13982 - 37366) | 15.05(11.67 - 20.44) | 14.52(9.31 - 23.99) | -0.02 (-0.27 to 0.23) |
| Cambodia | 1431(945 - 1874) | 4459(2844 - 6152) | 27.23(18.27 - 35.43) | 33.14(21.33 - 45.39) | 0.65 (0.57 to 0.74) |
| Bhutan | 54(35 - 75) | 111(73 - 173) | 15.54(10.34 - 22.22) | 16.31(10.91 - 24.8) | 0.15 (0.06 to 0.24) |
| Pakistan | 16101(12902 - 20531) | 50302(36509 - 67210) | 22.88(18.83 - 29.03) | 30.24(22.56 - 39.58) | 0.92 (0.81 to 1.03) |
| Nepal | 1744(1215 - 2470) | 4465(3038 - 6631) | 14.16(10.09 - 20.24) | 16.91(11.7 - 24.57) | 0.59 (0.47 to 0.7) |

DALYs = Disability-Adjusted Life Years. ASDR = Age-standardised DALYs rate. AAPC = Average annual percentage change. UI, Uncertainty Interval. CI, confidence interval.
