# Supplementary material for: Space-time analysis of head and neck cancer in Asia and its 34 countries and territories (1990–2021): Implications from the Global Burden of Disease Study 2021
Source: PLoS One. 2025 Jun 17;20(6):e0326177. doi: 10.1371/journal.pone.0326177 (PMC12173354; doi:10.1371/journal.pone.0326177)
Supplement: S8 Table — (DOCX) [file pone.0326177.s008.docx]

**S8 Table.** DALYs of larynx cancer in 1990 and 2021, and their average annual percentage changes from 1990 to 2021.

| **Location** | **Number of DALYs in 1990 (95%UI)** | **Number of DALYs in 2021 (95%UI)** | **ASDR in 1990 (per 100,000 population,95%UI)** | **ASDR in 2021 (per 100,000 population, 95%UI)** | **AAPC of ASDR (95%CI)** |
| --- | --- | --- | --- | --- | --- |
| High-income Asia Pacific | 42367(36682 - 47460) | 31591(27473 - 35245) | 20.57(17.86 - 23.02) | 7.1(6.22 - 7.97) | -3.37 (-3.64 to -3.09) |
| East Asia | 372617(306395 - 438644) | 508207(397896 - 639686) | 39.94(33.02 - 46.95) | 22.59(17.73 - 28.27) | -1.88 (-2.04 to -1.72) |
| Southeast Asia | 93620(81149 - 105815) | 194228(167983 - 230361) | 33.9(29.46 - 38.31) | 27.61(23.9 - 32.69) | -0.65 (-0.76 to -0.55) |
| Central Asia | 42705(40929 - 44722) | 29422(26003 - 33078) | 83.67(80.18 - 87.67) | 32.31(28.65 - 36.25) | -3.12 (-3.39 to -2.85) |
| South Asia | 575770(490462 - 672477) | 1066560(929501 - 1228037) | 88.93(75.45 - 103.97) | 67.29(58.66 - 77.4) | -0.88 (-1.06 to -0.7) |
| Republic of Korea | 18154(12777 - 23075) | 11113(8265 - 14291) | 57.12(40.47 - 72.48) | 11.7(8.7 - 15.01) | -5.01 (-5.27 to -4.75) |
| Japan | 23295(22362 - 24202) | 19627(17846 - 20996) | 13.51(12.95 - 14.04) | 5.51(5.13 - 5.87) | -2.9 (-3.25 to -2.56) |
| Taiwan (Province of China) | 5246(4892 - 5647) | 6459(5760 - 7176) | 30.72(28.69 - 33.03) | 15.62(13.94 - 17.36) | -2.15 (-2.7 to -1.6) |
| Singapore | 869(785 - 954) | 786(685 - 893) | 38.1(34.3 - 41.9) | 9.07(7.91 - 10.24) | -4.53 (-5.26 to -3.8) |
| Brunei Darussalam | 49(38 - 60) | 64(50 - 84) | 47.15(36.77 - 59.4) | 17.89(13.98 - 22.99) | -3.11 (-3.36 to -2.85) |
| Malaysia | 3825(2955 - 4639) | 9080(7203 - 10874) | 39.54(30.27 - 48.36) | 30.68(24.25 - 36.8) | -0.97 (-1.21 to -0.73) |
| Seychelles | 77(66 - 91) | 126(102 - 153) | 137.92(116.9 - 162.74) | 100.72(81.77 - 121.39) | -1.05 (-1.3 to -0.79) |
| Kazakhstan | 13412(12369 - 14443) | 6666(5728 - 7626) | 96.4(88.91 - 103.84) | 33.24(28.57 - 38.05) | -3.45 (-3.92 to -2.98) |
| Mauritius | 418(392 - 453) | 716(648 - 772) | 53.8(50.4 - 58.18) | 37.82(34.29 - 40.6) | -1.25 (-3.09 to 0.62) |
| Georgia | 7679(7052 - 8339) | 4807(4165 - 5502) | 118(108.32 - 128.2) | 84.43(73.11 - 96.63) | -0.54 (-1.07 to -0.01) |
| Sri Lanka | 2622(2108 - 3319) | 6483(3783 - 9927) | 22.85(18.48 - 28.95) | 23.29(13.7 - 35.5) | 0.08 (-0.43 to 0.59) |
| Armenia | 3544(3363 - 3691) | 1995(1783 - 2242) | 116.23(110.2 - 121.07) | 46.08(41.17 - 51.83) | -3.08 (-3.94 to -2.2) |
| Thailand | 19802(16000 - 23818) | 35607(25971 - 46962) | 51.35(41.52 - 61.54) | 33(24.19 - 43.18) | -1.42 (-1.66 to -1.17) |
| China | 362503(295796 - 428646) | 493848(382572 - 626010) | 40.37(33.13 - 47.6) | 22.73(17.67 - 28.65) | -1.89 (-2.06 to -1.73) |
| Azerbaijan | 4937(4303 - 5711) | 5766(4248 - 8306) | 87.95(76.03 - 101.65) | 48.69(35.87 - 69.88) | -1.86 (-2.07 to -1.66) |
| Turkmenistan | 1541(1432 - 1651) | 1385(1059 - 1827) | 71.89(66.77 - 76.98) | 29.87(22.96 - 39.32) | -3.32 (-3.71 to -2.92) |
| Indonesia | 30390(23072 - 36886) | 65905(47528 - 87716) | 28.01(21.1 - 34.11) | 25.21(18.01 - 33.31) | -0.34 (-0.38 to -0.3) |
| Uzbekistan | 7827(6775 - 9022) | 5341(4151 - 6849) | 63.01(54.78 - 72.37) | 17.79(14.03 - 22.45) | -4.03 (-4.92 to -3.13) |
| Philippines | 7959(6672 - 9616) | 19062(15250 - 23110) | 24.31(20.3 - 29.64) | 21.43(17.18 - 25.84) | -0.44 (-0.5 to -0.37) |
| Viet Nam | 14260(10969 - 18767) | 38867(27910 - 52932) | 34.43(26.62 - 45.17) | 35.88(26.06 - 48.19) | 0.15 (0.06 to 0.24) |
| Mongolia | 472(334 - 622) | 625(441 - 873) | 41.58(29.7 - 54.62) | 22.36(16.27 - 30.75) | -1.98 (-2.44 to -1.52) |
| Kyrgyzstan | 1912(1595 - 2270) | 1150(852 - 1469) | 59.83(49.89 - 71.41) | 20.78(15.43 - 26.43) | -3.35 (-4.41 to -2.27) |
| India | 434765(356863 - 520651) | 801557(690615 - 926887) | 82.09(66.95 - 98.23) | 62.95(54.37 - 72.82) | -0.86 (-1.1 to -0.61) |
| Maldives | 28(19 - 37) | 39(28 - 52) | 29.57(21.07 - 38.03) | 10.92(8.04 - 14.24) | -3.3 (-3.55 to -3.04) |
| Democratic People's Republic of Korea | 4868(3422 - 6608) | 7901(5396 - 10695) | 26.8(19.1 - 36.06) | 22.83(15.75 - 30.45) | -0.51 (-0.57 to -0.46) |
| Tajikistan | 1380(1019 - 1750) | 1688(1125 - 2412) | 46.06(34.15 - 58.65) | 24.04(16.28 - 33.99) | -2.17 (-2.48 to -1.85) |
| Myanmar | 10685(6529 - 14871) | 11481(7982 - 16353) | 42.76(27.02 - 58.98) | 22.31(15.71 - 31.62) | -2.09 (-2.16 to -2.01) |
| Timor-Leste | 84(56 - 123) | 204(142 - 293) | 26.54(18.33 - 37.84) | 23(15.98 - 32.85) | -0.44 (-0.67 to -0.21) |
| Lao People's Democratic Republic | 1048(723 - 1471) | 1422(950 - 2113) | 46.51(32.32 - 64.77) | 28.53(19.44 - 41.98) | -1.55 (-1.6 to -1.5) |
| Bangladesh | 52907(40938 - 66116) | 81431(57310 - 112543) | 105.03(81.68 - 131.7) | 56.28(39.91 - 77.04) | -1.9 (-2.14 to -1.67) |
| Cambodia | 2284(1675 - 3067) | 4965(3447 - 7526) | 46.81(34.75 - 62.47) | 37.26(25.97 - 56.2) | -0.7 (-0.83 to -0.56) |
| Bhutan | 188(121 - 273) | 300(205 - 418) | 67.45(43.78 - 97.39) | 47.62(32.68 - 66.34) | -1.11 (-1.21 to -1.01) |
| Pakistan | 79633(64276 - 97884) | 169489(124983 - 227793) | 132.92(107.23 - 163.79) | 121.47(88.73 - 161.68) | -0.28 (-0.36 to -0.21) |
| Nepal | 8277(5591 - 11655) | 13783(9970 - 19375) | 79.08(54.02 - 110.21) | 55.92(40.68 - 78.51) | -1.09 (-1.21 to -0.98) |

DALYs = Disability-Adjusted Life Years. ASDR = Age-standardised DALYs rate. AAPC = Average annual percentage change. UI, Uncertainty Interval. CI, confidence interval.
