# Supplementary material for: Space-time analysis of head and neck cancer in Asia and its 34 countries and territories (1990–2021): Implications from the Global Burden of Disease Study 2021
Source: PLoS One. 2025 Jun 17;20(6):e0326177. doi: 10.1371/journal.pone.0326177 (PMC12173354; doi:10.1371/journal.pone.0326177)
Supplement: S9 Table — (DOCX) [file pone.0326177.s009.docx]

**S9 Table.** DALYs of lip and oral cavity cancer in 1990 and 2021, and their average annual percentage changes from 1990 to 2021.

| **Location** | **Number of DALYs in 1990 (95%UI)** | **Number of DALYs in 2021 (95%UI)** | **ASDR in 1990 (per 100,000 population,95%UI)** | **ASDR in 2021 (per 100,000 population, 95%UI)** | **AAPC of ASDR (95%CI)** |
| --- | --- | --- | --- | --- | --- |
| High-income Asia Pacific | 53404(51574 - 55281) | 108025(96340 - 116039) | 26.22(25.27 - 27.15) | 26.82(24.59 - 28.51) | 0 (-0.34 to 0.34) |
| East Asia | 315772(267868 - 362040) | 697684(563155 - 853798) | 32.96(28.12 - 37.65) | 31.88(25.83 - 38.82) | -0.12 (-0.33 to 0.09) |
| Southeast Asia | 178289(154261 - 200931) | 422231(365828 - 481880) | 63.4(55.13 - 71.04) | 60.97(52.95 - 69.5) | -0.13 (-0.18 to -0.07) |
| Central Asia | 27174(25373 - 29368) | 38199(33257 - 43856) | 53.46(49.82 - 57.84) | 42.26(36.89 - 48.31) | -0.68 (-1.1 to -0.25) |
| South Asia | 1185410(1073536 - 1311139) | 2964855(2541835 - 3330176) | 174.46(156.86 - 192.99) | 182.29(157.06 - 203.68) | 0.17 (0.06 to 0.28) |
| Republic of Korea | 9257(8141 - 10727) | 17767(13735 - 21425) | 26.94(23.7 - 31.1) | 20.04(15.51 - 24.24) | -0.95 (-1.11 to -0.79) |
| Japan | 43216(41616 - 44394) | 88224(77814 - 94400) | 25.86(24.85 - 26.58) | 29.37(27.28 - 30.71) | 0.32 (-0.17 to 0.81) |
| Taiwan (Province of China) | 14650(13432 - 15778) | 69035(62158 - 76688) | 82.07(75.34 - 88.21) | 178.11(160.54 - 197.74) | 2.54 (2.16 to 2.94) |
| Singapore | 826(760 - 895) | 1794(1641 - 1966) | 33.45(30.73 - 36.11) | 21.03(19.17 - 23.08) | -1.37 (-2.46 to -0.26) |
| Brunei Darussalam | 105(85 - 131) | 240(195 - 290) | 78.14(63.68 - 95.73) | 58.27(47.83 - 70.37) | -0.95 (-1.13 to -0.77) |
| Malaysia | 8046(6741 - 9459) | 21065(17561 - 25329) | 78.62(66.11 - 92.06) | 70.76(59.2 - 84.83) | -0.44 (-0.64 to -0.24) |
| Seychelles | 84(71 - 99) | 202(170 - 238) | 149.86(126.13 - 177.44) | 162.75(137.26 - 189.83) | 0.27 (0.11 to 0.43) |
| Kazakhstan | 10702(9419 - 12107) | 10504(8679 - 12532) | 77.26(67.86 - 87.44) | 54.02(44.68 - 64.33) | -1.26 (-1.99 to -0.53) |
| Mauritius | 580(548 - 614) | 1180(1085 - 1253) | 74.95(71.02 - 79.46) | 64.42(59.47 - 68.28) | -0.42 (-1.81 to 1) |
| Georgia | 2872(2436 - 3347) | 3409(2947 - 3899) | 45.26(38.45 - 52.57) | 62.02(53.83 - 70.73) | 1.13 (0.1 to 2.18) |
| Sri Lanka | 12466(10711 - 14390) | 30079(18687 - 43223) | 110.05(94.47 - 127.56) | 110.56(69.19 - 157.92) | 0.23 (-0.13 to 0.6) |
| Armenia | 810(684 - 957) | 1238(1027 - 1457) | 27.23(22.99 - 32.12) | 29.15(24.16 - 34.35) | 0.23 (-0.28 to 0.74) |
| Thailand | 37393(31265 - 43965) | 83054(63789 - 105555) | 95.4(80.28 - 111.64) | 79.71(61.59 - 100.69) | -0.59 (-0.69 to -0.48) |
| China | 295390(247607 - 342342) | 618016(487509 - 777184) | 32.09(27.04 - 37.01) | 29.2(23.18 - 36.49) | -0.32 (-0.48 to -0.16) |
| Azerbaijan | 1144(797 - 1618) | 2117(1319 - 3237) | 20.84(14.7 - 29.31) | 18.57(11.93 - 27.9) | -0.36 (-0.63 to -0.09) |
| Turkmenistan | 1624(1420 - 1822) | 3035(2283 - 4102) | 75.03(65.5 - 84.42) | 65.88(49.88 - 88.57) | -0.15 (-0.75 to 0.45) |
| Indonesia | 48076(36745 - 58870) | 119345(87051 - 151634) | 43.12(32.65 - 52.42) | 46.19(33.93 - 58.01) | 0.23 (0.19 to 0.27) |
| Uzbekistan | 4876(4008 - 5830) | 13043(10445 - 16014) | 38.56(31.43 - 46.94) | 43.19(34.25 - 53.5) | 0.35 (-0.19 to 0.91) |
| Philippines | 18524(15252 - 21227) | 42491(35652 - 49938) | 54.32(43.85 - 62.72) | 47.25(39.92 - 55.29) | -0.45 (-0.55 to -0.35) |
| Viet Nam | 34968(26773 - 44267) | 89683(66736 - 115436) | 82.94(63.88 - 104.82) | 83.23(63.08 - 106.35) | 0.02 (-0.03 to 0.08) |
| Mongolia | 895(636 - 1233) | 1281(910 - 1711) | 77.2(54.94 - 106.79) | 46.7(33 - 62.2) | -1.62 (-1.88 to -1.36) |
| Kyrgyzstan | 3518(3003 - 4044) | 2424(1984 - 2955) | 111.54(95.3 - 127.83) | 44.08(36.46 - 53.81) | -3.13 (-3.83 to -2.43) |
| India | 848710(758330 - 946701) | 2118894(1793575 - 2398412) | 152.94(135.81 - 169.35) | 163.61(139.58 - 184.48) | 0.22 (0.06 to 0.37) |
| Maldives | 64(42 - 96) | 154(116 - 194) | 68.34(49.07 - 96.25) | 41.48(31.65 - 52.25) | -1.7 (-1.93 to -1.46) |
| Democratic People's Republic of Korea | 5732(4110 - 7928) | 10634(7270 - 14737) | 31.26(22.49 - 42.59) | 31.13(21.66 - 42.91) | -0.01 (-0.04 to 0.02) |
| Tajikistan | 732(530 - 969) | 1148(808 - 1583) | 24.99(17.98 - 33.01) | 16.78(12.02 - 22.7) | -1.29 (-1.54 to -1.04) |
| Myanmar | 13460(8898 - 21299) | 22803(16363 - 30830) | 53.01(36.16 - 82.08) | 44.41(32.17 - 59.9) | -0.59 (-0.64 to -0.54) |
| Timor-Leste | 136(92 - 182) | 383(279 - 508) | 40.57(27.57 - 53.42) | 42.64(30.99 - 56.59) | 0.15 (0.02 to 0.27) |
| Lao People's Democratic Republic | 1308(887 - 1993) | 2583(1865 - 3336) | 57.48(39.59 - 85.93) | 50.07(36.38 - 64.22) | -0.43 (-0.5 to -0.37) |
| Bangladesh | 94512(66731 - 117823) | 187496(117216 - 266032) | 175.94(123.53 - 219.39) | 127.46(80.66 - 179.96) | -1 (-1.14 to -0.87) |
| Cambodia | 2927(2041 - 4072) | 8620(6194 - 11851) | 58.58(42.08 - 80.14) | 64.54(47.2 - 86.75) | 0.33 (0.28 to 0.39) |
| Bhutan | 449(284 - 622) | 803(541 - 1156) | 148.84(95.74 - 206.62) | 122.12(83.91 - 173.03) | -0.63 (-0.71 to -0.55) |
| Pakistan | 226497(182385 - 275046) | 625099(485940 - 802124) | 357.93(290.69 - 433.42) | 410.22(321.29 - 518.79) | 0.45 (0.36 to 0.54) |
| Nepal | 15241(11276 - 21086) | 32564(23406 - 44115) | 138.97(101.37 - 190.15) | 129.18(94.08 - 173.87) | -0.23 (-0.31 to -0.14) |

DALYs = Disability-Adjusted Life Years. ASDR = Age-standardised DALYs rate. AAPC = Average annual percentage change. UI, Uncertainty Interval. CI, confidence interval.
