# Supplementary material for: Space-time analysis of head and neck cancer in Asia and its 34 countries and territories (1990–2021): Implications from the Global Burden of Disease Study 2021
Source: PLoS One. 2025 Jun 17;20(6):e0326177. doi: 10.1371/journal.pone.0326177 (PMC12173354; doi:10.1371/journal.pone.0326177)
Supplement: S10 Table — (DOCX) [file pone.0326177.s010.docx]

**S10 Table.** DALYs of other pharynx cancer in 1990 and 2021, and their average annual percentage changes from 1990 to 2021.

| **Location** | **Number of DALYs in 1990 (95%UI)** | **Number of DALYs in 2021 (95%UI)** | **ASDR in 1990 (per 100,000 population,95%UI)** | **ASDR in 2021 (per 100,000 population, 95%UI)** | **AAPC of ASDR (95%CI)** |
| --- | --- | --- | --- | --- | --- |
| High-income Asia Pacific | 24214(23381 - 25062) | 78591(72884 - 83220) | 11.56(11.16 - 11.96) | 19.47(18.14 - 20.61) | 1.75 (1.57 to 1.94) |
| East Asia | 136914(112557 - 166109) | 185965(150251 - 229687) | 14.24(11.76 - 17.21) | 8.33(6.78 - 10.21) | -1.74 (-2.02 to -1.46) |
| Southeast Asia | 58567(49243 - 68732) | 144945(120816 - 172263) | 20.42(17.16 - 23.98) | 19.96(16.63 - 23.67) | -0.09 (-0.16 to -0.01) |
| Central Asia | 12827(11764 - 14069) | 15698(13556 - 18181) | 25.04(22.95 - 27.46) | 17(14.72 - 19.63) | -1.31 (-2 to -0.61) |
| South Asia | 547876(463107 - 646548) | 1511059(1320727 - 1703791) | 80.64(67.81 - 95.51) | 93(81.35 - 104.74) | 0.49 (0.34 to 0.63) |
| Republic of Korea | 3427(2866 - 4069) | 11274(8551 - 14124) | 10.12(8.48 - 12.06) | 11.87(9.09 - 14.69) | 0.51 (0.38 to 0.63) |
| Japan | 20332(19661 - 20936) | 66005(61225 - 68963) | 11.71(11.32 - 12.06) | 22.39(21.17 - 23.28) | 2.14 (1.92 to 2.37) |
| Taiwan (Province of China) | 6003(5562 - 6446) | 27798(24512 - 30696) | 34.41(31.79 - 37.16) | 70.69(62.67 - 77.73) | 2.34 (1.71 to 2.97) |
| Singapore | 418(355 - 478) | 1187(1029 - 1387) | 17.27(14.72 - 19.75) | 13.39(11.62 - 15.64) | -0.55 (-1.31 to 0.22) |
| Brunei Darussalam | 38(29 - 49) | 125(103 - 152) | 33.63(25.29 - 43.31) | 30.6(25.05 - 37) | -0.35 (-0.65 to -0.05) |
| Malaysia | 2435(2007 - 2928) | 6596(5430 - 7996) | 24.01(19.81 - 29.03) | 21.64(17.82 - 26.16) | -0.3 (-0.59 to 0) |
| Seychelles | 39(32 - 47) | 107(86 - 129) | 70.49(56.94 - 85.81) | 83.21(66.89 - 100.02) | 0.54 (0.2 to 0.88) |
| Kazakhstan | 5874(5172 - 6629) | 3889(3201 - 4736) | 42(36.98 - 47.48) | 19.53(16.08 - 23.74) | -2.51 (-3.55 to -1.45) |
| Mauritius | 65(60 - 69) | 310(283 - 337) | 8.17(7.62 - 8.73) | 16.42(14.96 - 17.75) | 1.43 (-1.31 to 4.25) |
| Georgia | 1414(1082 - 1884) | 1531(1318 - 1748) | 22.09(17.01 - 29.46) | 27.68(23.87 - 31.59) | 0.93 (-0.11 to 1.98) |
| Sri Lanka | 4930(3979 - 6104) | 7454(4516 - 10956) | 42.11(34.28 - 52.17) | 26.84(16.21 - 39.31) | -1.37 (-2.04 to -0.69) |
| Armenia | 326(264 - 407) | 436(358 - 517) | 10.65(8.62 - 13.28) | 10.11(8.28 - 12.01) | -0.61 (-1.25 to 0.03) |
| Thailand | 8276(6299 - 10747) | 18110(13253 - 24942) | 20.14(15.41 - 25.93) | 16.94(12.46 - 23.09) | -0.49 (-0.71 to -0.27) |
| China | 129153(104815 - 158416) | 155670(122186 - 199433) | 13.96(11.4 - 17.06) | 7.21(5.69 - 9.16) | -2.17 (-2.45 to -1.89) |
| Azerbaijan | 550(354 - 797) | 928(571 - 1438) | 9.84(6.4 - 14.43) | 7.85(4.85 - 12.06) | -0.75 (-1.04 to -0.45) |
| Turkmenistan | 883(780 - 986) | 1448(1077 - 1971) | 40.63(35.92 - 45.39) | 30.86(23.1 - 41.78) | -0.86 (-1.45 to -0.27) |
| Indonesia | 13599(10246 - 17799) | 30697(21169 - 41877) | 12(8.99 - 15.88) | 11.4(7.89 - 15.58) | -0.16 (-0.22 to -0.11) |
| Uzbekistan | 2426(1780 - 3200) | 5236(3833 - 6845) | 19.22(14.18 - 25.65) | 16.97(12.7 - 21.75) | -0.36 (-1.36 to 0.66) |
| Philippines | 3846(3267 - 4676) | 9894(7930 - 12114) | 11.15(9.44 - 13.68) | 10.85(8.76 - 13.3) | -0.05 (-0.13 to 0.03) |
| Viet Nam | 20380(14779 - 27409) | 63135(45728 - 83645) | 48.41(35.22 - 64.94) | 56.74(41.49 - 74.62) | 0.54 (0.41 to 0.66) |
| Mongolia | 301(218 - 419) | 447(326 - 606) | 25.97(18.61 - 36.42) | 15.68(11.47 - 21.2) | -1.68 (-2.07 to -1.28) |
| Kyrgyzstan | 525(408 - 654) | 943(722 - 1207) | 16.58(12.99 - 20.62) | 16.81(12.99 - 21.43) | 0.11 (-0.51 to 0.74) |
| India | 442152(370021 - 532320) | 1242130(1075828 - 1416056) | 79.68(66.69 - 95.88) | 95.27(82.55 - 108.59) | 0.61 (0.43 to 0.78) |
| Maldives | 18(11 - 26) | 44(32 - 58) | 18.42(12.21 - 25.21) | 11.22(8.36 - 14.91) | -1.63 (-2.16 to -1.1) |
| Democratic People's Republic of Korea | 1758(1257 - 2407) | 2497(1757 - 3388) | 9.4(6.77 - 12.68) | 7.2(5.13 - 9.72) | -0.86 (-0.89 to -0.83) |
| Tajikistan | 528(379 - 753) | 841(565 - 1155) | 17.78(12.96 - 24.91) | 11.73(8.13 - 16.16) | -1.36 (-1.8 to -0.92) |
| Myanmar | 3684(2570 - 5427) | 5602(3935 - 7876) | 14.32(10.13 - 21.11) | 10.66(7.54 - 14.95) | -0.95 (-1 to -0.9) |
| Timor-Leste | 36(23 - 51) | 87(61 - 125) | 10.42(6.93 - 14.72) | 9.64(6.72 - 13.71) | -0.27 (-0.53 to 0) |
| Lao People's Democratic Republic | 383(253 - 573) | 644(447 - 887) | 16.53(11.06 - 24.57) | 12.31(8.69 - 16.88) | -0.94 (-0.98 to -0.89) |
| Bangladesh | 50839(35687 - 68556) | 118310(74487 - 190043) | 97.3(68.24 - 131.47) | 80.11(50.47 - 127.72) | -0.54 (-0.74 to -0.33) |
| Cambodia | 791(593 - 1086) | 2064(1479 - 2855) | 15.6(11.81 - 21.09) | 15.07(10.91 - 20.66) | -0.1 (-0.16 to -0.04) |
| Bhutan | 227(141 - 341) | 492(345 - 698) | 76.85(48.96 - 115.49) | 75.89(53.54 - 107.13) | -0.04 (-0.09 to 0) |
| Pakistan | 46844(38165 - 57040) | 130925(98365 - 175028) | 75.65(61.33 - 92.09) | 88.36(66.74 - 116.72) | 0.52 (0.44 to 0.61) |
| Nepal | 7814(5679 - 10769) | 19203(13933 - 26462) | 71.09(51.61 - 97.63) | 75.81(55.22 - 104.71) | 0.23 (0.08 to 0.37) |

DALYs = Disability-Adjusted Life Years. ASDR = Age-standardised DALYs rate. AAPC = Average annual percentage change. UI, Uncertainty Interval. CI, confidence interval.
