# Supplementary material for: Space-time analysis of head and neck cancer in Asia and its 34 countries and territories (1990–2021): Implications from the Global Burden of Disease Study 2021
Source: PLoS One. 2025 Jun 17;20(6):e0326177. doi: 10.1371/journal.pone.0326177 (PMC12173354; doi:10.1371/journal.pone.0326177)
Supplement: S11 Table — (DOCX) [file pone.0326177.s011.docx]

**S11 Table.** Projections of DALYs and age-standardized DALYs rates for head and neck cancers and it’s five types (nasopharynx cancer, thyroid cancer, larynx cancer, lip and oral cavity cancer, and other pharynx cancer) from 2022 to 2030 at 5 Asia regions.

| **Cause** | **Year** | **High-income Asia Pacific** | | **East Asia** | | **South Asia** | | **Central Asia** | | **Southeast Asia** | |
| --- | --- | --- | --- | --- | --- | --- | --- | --- | --- | --- | --- |
|  |  | **DALYs** | **ASDR** | **DALYs** | **ASDR** | **DALYs** | **ASDR** | **DALYs** | **ASDR** | **DALYs** | **ASDR** |
| Head and neck cancer | 2022 | 138,462 | 74.39 | 1,890,027 | 125.67 | 8,026,316 | 394.36 | 108,963 | 112.54 | 1,283,617 | 185.33 |
| Head and neck cancer | 2023 | 137,151 | 73.81 | 1,891,660 | 125.66 | 8,087,555 | 394.4 | 107,510 | 109.8 | 1,297,146 | 185.63 |
| Head and neck cancer | 2024 | 135,805 | 73.22 | 1,891,753 | 125.61 | 8,146,233 | 394.43 | 106,060 | 107.17 | 1,310,136 | 185.89 |
| Head and neck cancer | 2025 | 134,430 | 72.63 | 1,890,182 | 125.52 | 8,202,187 | 394.47 | 104,664 | 104.68 | 1,322,360 | 186.09 |
| Head and neck cancer | 2026 | 133,031 | 72.04 | 1,886,973 | 125.37 | 8,255,382 | 394.54 | 103,334 | 102.34 | 1,333,906 | 186.24 |
| Head and neck cancer | 2027 | 131,599 | 71.45 | 1,882,388 | 125.19 | 8,306,137 | 394.62 | 101,990 | 100.06 | 1,344,889 | 186.35 |
| Head and neck cancer | 2028 | 130,135 | 70.85 | 1,876,442 | 124.97 | 8,353,257 | 394.66 | 100,607 | 97.81 | 1,355,296 | 186.44 |
| Head and neck cancer | 2029 | 128,643 | 70.26 | 1,868,990 | 124.7 | 8,396,657 | 394.64 | 99,228 | 95.62 | 1,365,032 | 186.48 |
| Head and neck cancer | 2030 | 127,127 | 69.66 | 1,859,868 | 124.38 | 8,436,762 | 394.59 | 97,916 | 93.53 | 1,373,880 | 186.45 |
| Nasopharynx cancer | 2022 | 15,719 | 8.44 | 752,690 | 50.05 | 662,570 | 32.55 | 12,386 | 12.79 | 353,770 | 51.08 |
| Nasopharynx cancer | 2023 | 15,446 | 8.31 | 754,444 | 50.12 | 663,205 | 32.34 | 12,292 | 12.55 | 356,365 | 51 |
| Nasopharynx cancer | 2024 | 15,167 | 8.18 | 755,614 | 50.17 | 663,489 | 32.13 | 12,190 | 12.32 | 358,794 | 50.91 |
| Nasopharynx cancer | 2025 | 14,888 | 8.04 | 756,105 | 50.21 | 663,457 | 31.91 | 12,084 | 12.09 | 360,995 | 50.8 |
| Nasopharynx cancer | 2026 | 14,609 | 7.91 | 755,871 | 50.22 | 663,165 | 31.69 | 11,973 | 11.86 | 362,971 | 50.68 |
| Nasopharynx cancer | 2027 | 14,327 | 7.78 | 755,041 | 50.22 | 662,622 | 31.48 | 11,857 | 11.63 | 364,744 | 50.54 |
| Nasopharynx cancer | 2028 | 14,043 | 7.65 | 753,668 | 50.19 | 661,709 | 31.26 | 11,736 | 11.41 | 366,328 | 50.39 |
| Nasopharynx cancer | 2029 | 13,756 | 7.51 | 751,675 | 50.15 | 660,405 | 31.04 | 11,612 | 11.19 | 367,719 | 50.23 |
| Nasopharynx cancer | 2030 | 13,470 | 7.38 | 748,936 | 50.08 | 658,786 | 30.81 | 11,487 | 10.97 | 368,861 | 50.06 |
| Thyroid cancer | 2022 | 22,103 | 11.87 | 153,781 | 10.23 | 368,741 | 18.12 | 11,078 | 11.44 | 164,565 | 23.76 |
| Thyroid cancer | 2023 | 21,916 | 11.79 | 153,342 | 10.19 | 371,545 | 18.12 | 10,975 | 11.21 | 166,263 | 23.79 |
| Thyroid cancer | 2024 | 21,729 | 11.72 | 152,838 | 10.15 | 374,175 | 18.12 | 10,863 | 10.98 | 167,908 | 23.82 |
| Thyroid cancer | 2025 | 21,540 | 11.64 | 152,247 | 10.11 | 376,640 | 18.11 | 10,760 | 10.76 | 169,474 | 23.85 |
| Thyroid cancer | 2026 | 21,350 | 11.56 | 151,573 | 10.07 | 378,894 | 18.11 | 10,656 | 10.55 | 170,951 | 23.87 |
| Thyroid cancer | 2027 | 21,158 | 11.49 | 150,853 | 10.03 | 380,948 | 18.1 | 10,540 | 10.34 | 172,349 | 23.88 |
| Thyroid cancer | 2028 | 20,963 | 11.41 | 150,107 | 10 | 382,765 | 18.08 | 10,413 | 10.12 | 173,681 | 23.89 |
| Thyroid cancer | 2029 | 20,765 | 11.34 | 149,316 | 9.96 | 384,343 | 18.06 | 10,282 | 9.91 | 174,946 | 23.9 |
| Thyroid cancer | 2030 | 20,564 | 11.27 | 148,457 | 9.93 | 385,721 | 18.04 | 10,161 | 9.71 | 176,116 | 23.9 |
| Larynx cancer | 2022 | 13,040 | 7.01 | 333,190 | 22.15 | 1,340,303 | 65.85 | 29,540 | 30.51 | 190,551 | 27.51 |
| Larynx cancer | 2023 | 12,908 | 6.95 | 329,049 | 21.86 | 1,342,408 | 65.46 | 28,841 | 29.46 | 191,440 | 27.4 |
| Larynx cancer | 2024 | 12,771 | 6.89 | 324,783 | 21.57 | 1,344,205 | 65.08 | 28,139 | 28.43 | 192,250 | 27.28 |
| Larynx cancer | 2025 | 12,628 | 6.82 | 320,386 | 21.27 | 1,345,556 | 64.71 | 27,453 | 27.46 | 192,911 | 27.15 |
| Larynx cancer | 2026 | 12,479 | 6.76 | 315,877 | 20.99 | 1,346,504 | 64.35 | 26,793 | 26.54 | 193,425 | 27.01 |
| Larynx cancer | 2027 | 12,321 | 6.69 | 311,312 | 20.7 | 1,347,119 | 64 | 26,136 | 25.64 | 193,838 | 26.86 |
| Larynx cancer | 2028 | 12,155 | 6.62 | 306,716 | 20.43 | 1,347,343 | 63.66 | 25,478 | 24.77 | 194,182 | 26.71 |
| Larynx cancer | 2029 | 11,986 | 6.55 | 302,075 | 20.15 | 1,347,169 | 63.32 | 24,820 | 23.92 | 194,438 | 26.56 |
| Larynx cancer | 2030 | 11,813 | 6.47 | 297,376 | 19.89 | 1,346,606 | 62.98 | 24,182 | 23.1 | 194,545 | 26.4 |
| Lip and oral cavity cancer | 2022 | 50,136 | 26.93 | 486,125 | 32.32 | 3,718,043 | 182.68 | 40,222 | 41.54 | 424,978 | 61.36 |
| Lip and oral cavity cancer | 2023 | 49,741 | 26.77 | 484,197 | 32.17 | 3,751,748 | 182.96 | 40,027 | 40.88 | 429,531 | 61.47 |
| Lip and oral cavity cancer | 2024 | 49,343 | 26.6 | 482,020 | 32.01 | 3,784,746 | 183.25 | 39,828 | 40.25 | 433,943 | 61.57 |
| Lip and oral cavity cancer | 2025 | 48,943 | 26.44 | 479,559 | 31.84 | 3,816,798 | 183.56 | 39,612 | 39.62 | 438,145 | 61.66 |
| Lip and oral cavity cancer | 2026 | 48,542 | 26.29 | 476,799 | 31.68 | 3,847,876 | 183.9 | 39,393 | 39.02 | 442,193 | 61.74 |
| Lip and oral cavity cancer | 2027 | 48,135 | 26.13 | 473,798 | 31.51 | 3,878,144 | 184.25 | 39,152 | 38.41 | 446,119 | 61.82 |
| Lip and oral cavity cancer | 2028 | 47,725 | 25.98 | 470,585 | 31.34 | 3,907,293 | 184.6 | 38,890 | 37.81 | 449,912 | 61.89 |
| Lip and oral cavity cancer | 2029 | 47,314 | 25.84 | 467,108 | 31.17 | 3,935,292 | 184.96 | 38,625 | 37.22 | 453,525 | 61.96 |
| Lip and oral cavity cancer | 2030 | 46,901 | 25.7 | 463,323 | 30.98 | 3,962,212 | 185.31 | 38,351 | 36.63 | 456,893 | 62 |
| Other pharynx cancer | 2022 | 35,655 | 19.15 | 125,156 | 8.32 | 1,889,437 | 92.83 | 16,204 | 16.74 | 138,597 | 20.01 |
| Other pharynx cancer | 2023 | 35,044 | 18.86 | 125,246 | 8.32 | 1,910,046 | 93.15 | 16,141 | 16.49 | 139,830 | 20.01 |
| Other pharynx cancer | 2024 | 34,419 | 18.56 | 125,297 | 8.32 | 1,930,298 | 93.46 | 16,076 | 16.24 | 141,001 | 20.01 |
| Other pharynx cancer | 2025 | 33,790 | 18.26 | 125,305 | 8.32 | 1,950,065 | 93.79 | 16,015 | 16.02 | 142,080 | 19.99 |
| Other pharynx cancer | 2026 | 33,167 | 17.96 | 125,271 | 8.32 | 1,969,407 | 94.12 | 15,957 | 15.8 | 143,076 | 19.98 |
| Other pharynx cancer | 2027 | 32,548 | 17.67 | 125,211 | 8.33 | 1,988,421 | 94.47 | 15,894 | 15.59 | 144,017 | 19.96 |
| Other pharynx cancer | 2028 | 31,942 | 17.39 | 125,134 | 8.33 | 2,006,855 | 94.82 | 15,824 | 15.38 | 144,900 | 19.93 |
| Other pharynx cancer | 2029 | 31,335 | 17.11 | 125,020 | 8.34 | 2,024,640 | 95.16 | 15,752 | 15.18 | 145,708 | 19.91 |
| Other pharynx cancer | 2030 | 30,737 | 16.84 | 124,851 | 8.35 | 2,041,844 | 95.5 | 15,688 | 14.99 | 146,418 | 19.87 |

ASDR = Age-standardised DALYs rate. DALYs = disabilityadjusted life-years.
