# Supplementary material for: Space-time analysis of head and neck cancer in Asia and its 34 countries and territories (1990–2021): Implications from the Global Burden of Disease Study 2021
Source: PLoS One. 2025 Jun 17;20(6):e0326177. doi: 10.1371/journal.pone.0326177 (PMC12173354; doi:10.1371/journal.pone.0326177)
Supplement: S1 Fig — (DOCX) [file pone.0326177.s012.docx]

**S1 Fig.** DALYs of head and neck cancer and its five subtypes across five Asia subregions in 1990 and 2021.
